# A Taxonomic and Phylogenetic Study of Anamorphic Strains of *Daldinia* (*Hypoxylaceae*, *Xylariales*) in Southern China

**DOI:** 10.3390/jof10100700

**Published:** 2024-10-07

**Authors:** Changzhun Yin, Zhaoxue Zhang, Shi Wang, Wenwen Liu, Xiuguo Zhang

**Affiliations:** 1 College of Life Sciences, Shandong Normal University, Jinan 250300, China; zcy94156@163.com (C.Y.); wangssdau@126.com (S.W.); 17615593869@163.com (W.L.); 2 Shandong Provincial Key Laboratory for Biology of Vegetable Diseases and Insect Pests, College of Plant Protection, Shandong Agricultural University, Taian 271000, China; zhangzhaoxue2022@126.com

**Keywords:** biodiversity, new species, morphology, phylogeny, taxonomy

## Abstract

In an extensive fungal investigation conducted in southern China, a large number of fungal strains were isolated by collecting and treating diseased and decayed leaves. Using internal transcribed spacer regions (ITSs) sequence data for a BLAST search to screen for suspected strains of *Daldinia*, followed by phylogenetic analysis using internal transcribed spacer regions, partial sequences of the large subunit of the rDNA (LSU), RNA polymerase II (*rpb2*), and beta tubulin (*tub2*) sequence data, combined with morphological characteristics of anamorphic species, ninety-four strains of *Daldinia* were identified. Furthermore, their geographical distribution and host specificity of the genus were thoroughly analyzed and summarized. Additionally, seven new anamorphic species of the genus *Daldinia* were also detected, *Daldinia ehretiae* sp. nov., *D. jianfengensis* sp. nov., *D. ledongensis* sp. nov., *D. menghaiensis* sp. nov., *D. rhododendri* sp. nov., *D. spatholobi* sp. nov., and *D. thunbergiae* sp. nov.

## 1. Introduction

The genus *Daldinia* (*Hypoxylaceae*, *Xylariales*) was introduced by Cesati and De Notaris, as a tribute to the Swiss monk, Agostino Daldini [1], and it is now recognized as one of the largest genera within *Hypoxylaceae* (*Xylariales*, *Ascomycota*). The identification of *Daldinia* species traditionally relies on the presence of internal concentric zones beneath the perithecial layer and the detection of KOH-extractable pigments below and on the stromatal surface [2]. The current common view is that *Daldinia* species usually inhabited dicots. However, there have been reports of *Daldinia bambusicola* isolated from bamboo (monocot) in Thailand, and records of isolated *Daldinia* from monocot plant hosts can even be traced back to the 19th century [2,3]. *Daldinia graminis* and *D. sacchari* were reported to occur on sugarcane in India [4]. The latest world monograph of *Daldinia* species has employed a combination of morphological, molecular phylogenetic characteristics, and chemical classification [3]. It has also compiled morphological, structural, and chemotaxonomic data for over a thousand cultures and specimens, including preliminary phylogenetic data based on ITS sequences. Notably, several *Daldinia* species, including *D. caldariorum*, *D. gelatinoides*, *D. loculata*, *D. loculatoides*, and *D. vernicosa*, have been documented to exhibit stromata production on wood after fire events [3]. In order to determine the phylogenetic relationships of *Daldinia* and its allied genera, the study conducted by Wendt et al. incorporated various species from different genera into a multi-locus phylogenetic analysis, revealing that *Daldinia* and its relatives form a distinct branch within the *Hypoxylaceae*, separate from *Hypoxylon* and *Pyrenopolyporus*, and Daranagama et al. presented a comprehensive analysis and introduction of the phylogeny for key taxa within *Xylariaceae*, accompanied by updated illustrations and descriptions of all taxa [5,6]. Wongkanoun et al. unveiled a new record and a new species of the genus *Daldinia* in northern Thailand by employing traditional morphological and a multi-locus phylogenetic analysis of ITS, LSU, *tub2*, and *rpb2* [7]. It is worth mentioning that Pažoutová et al. identified a new insect-associated, endophytic species, *Daldinia hawksworthii*, based on molecular data of anamorphic species and structural characteristics of their conidiophores, conidiogenous cells, and conidia, which provided a strong reference for our research [8].

China boasts abundant microbial resources across its vast territory, particularly in the southern regions characterized by intricate topography, diverse vegetation, and favorable climatic conditions. In this investigation, an extensive sampling effort was conducted across Yunnan, Hainan, Sichuan, Fujian, and Guizhou provinces of China to study fungal resources comprehensively. At present, there are few relevant articles on the systematic study of the genus *Daldinia* in China [3]. Therefore, the primary objective of this study was to comprehensively analyze the geographical distribution and host species exhibited by the genus *Daldinia* in southern China, as well as to provide detailed descriptions and explanations regarding the morphological and phylogenetic analysis of the anamorphic species of *Daldinia* encountered in this investigation.

## 2. Materials and Methods

### 2.1. Sample Treatment and Morphological Characterizations

Between 2022 and 2023, more than 6000 samples were collected from diseased and decayed leaves in densely vegetated areas such as forests and mountains in the Yunnan, Hainan, Sichuan, Guizhou, and Fujian provinces of China, based on the principle of collecting samples from different locations and plants as far as possible. Cut 3 × 3 mm small square leaves from the fungal infection site of the diseased and decayed leaves (the location selected for cutting is marked with a black circle and arrow in subsequent figure), disinfect the surface in 75% alcohol for 30 s, rinse once with sterile water, immerse in a 5% sodium hypochlorite solution for disinfection for 1 min, and finally rinse three times with sterile water. After disinfection, the small square leaves were dried on sterile filter paper and then transferred to the surface of Potato Dextrose Agar (PDA) (200 g potato, 20 g agar powder, 20 g dextrose, 1000 mL distilled water, and pH adjusted to 7.0) and Oatmeal Agar (OA) (25 g oats, 20 g agar powder, 1000 mL distilled water, and pH adjusted to 7.0). Three to five small square leaves can be placed on each medium; the strain number and date were marked on the medium and cultured in a constant temperature incubator at 25 °C, and the fungal growth was regularly observed. When there were more mycelia around the small square leaves, 5 × 5 mm agar blocks with mycelia were cut down with the inoculation needle and inoculated on the new PDA and OA medium and then placed in the incubator for culture. A digital camera (Canon Powershot G7X; Canon (China) Co., Ltd., Beijing, China) was utilized to capture mycelia growing on PDA medium and OA medium on day 7, day 14, and day 31, respectively. In addition, the color formed by the colonies during the cultivation process was recorded using the Pantone Colour Chart (https://www.pantone-colours.com/) (accessed on 20 June 2024). The asexual morphology of *Daldinia* was observed and recorded using a stereomicroscope (Olympus SZX10) and microscope (Olympus BX53). Additionally, a high-definition color digital camera (Olympus DP80) was employed to photograph the conidiogenous cells, conidia, and other structures. The microstructure of each strain (20–30 measurements per strain) was measured using the Digimizer v.5.4.7 (https://www.digimizer.com/) (accessed on 20 June 2024). All strains were stored in sterile 10% glycerol at 4 °C. Ex-type cultures were stored in the Shandong Agricultural University Culture Collection (SAUCC). Furthermore, voucher specimens were dried in the oven, sealed, and stored in the Herbarium Mycologicum Academiae Sinicae, Institute of Microbiology, Chinese Academy of Sciences, Beijing, China (HMAS), as well as in the Herbarium of the Department of Plant Pathology, Shandong Agricultural University, Taian, China (HSAUP). Taxonomic information on all new species of the genus *Daldinia* has been uploaded to MycoBank (http://www.mycobank.org/) (accessed on 20 June 2024).

### 2.2. DNA Extraction and Sequencing

The mycelium on PDA medium was subjected to the CTAB method and BeaverBeads Plant DNA Kit (Cat. No.: 70409-20; BEAVER Biomedical Engineering Co., Ltd., Suzhou, China) for genomic DNA extraction of each of the strains [9,10,11]. PCR amplification of the genomic DNA was performed using ITSs as primers. The reaction mixture (25 μL) consisted of 2 × Hieff Canace ^®^ Plus PCR Master Mix (Shanghai, China) (with dye) (Yeasen Bio-technology, Shanghai, China, Cat No. 10154ES03) (12 μL), 10 μmol/μL forward primer (1 μL), 10 μmol/μL reverse primer (1 μL), 10–20 μmol/μL genomic DNA (1 μL), and distilled deionized water (10 μL). The amplified products were detected on 2% agarose electrophoresis gel and the amplification effect was examined under UV light [12]. After the gel strips with the correct band were cut off, they were recycled through the Gel Extraction Kit (Cat: AE0101-C) (Shandong Sparkjade Biotechnology Co., Ltd., Jinan, China), and the obtained DNA solutions were sent to the biological company for sequencing. Primer synthesis and DNA sequencing were conducted by Tsingke Biotechnology Co., Ltd. (Qingdao, China). The obtained sequencing results were analyzed and assembled using MEGA v.7.0 [13]. The assembled ITS sequences were BLAST searched (https://blast.ncbi.nlm.nih.gov/) (accessed on 20 June 2024) to screen out suspected *Daldinia* strains, and then LSU, *rpb2*, and *tub2* primers were used to repeat the operation of the above ITS primers, and finally the sequences of ITS, LSU, *rpb2*, and *tub2* of all suspected strains were obtained. The primer sequences and reaction conditions used in this study are presented in the Supplementary Materials [14,15,16,17]. Finally, the sequences of all strains of the *Daldinia* genus have been uploaded to Genbank; GenBank accession numbers are shown in Table 1.

### 2.3. Phylogenetic Analyses

Based on the recently published literature on the genus *Daldinia*, the sequences for phylogenetic analysis of the *Daldinia* genus were downloaded from the National Center for Biotechnology Information (https://www.ncbi.nlm.nih.gov/) (accessed on 20 June 2024) [19]. The GenBank accession numbers of all sequences used in this experiment are shown in Table 1. All sequences for phylogenetic analysis were compared and manually corrected using MEGA v.7.0 [13]. The maximum likelihood (ML), Bayesian algorithm (MB), and maximum parsimony (MP) were used to analyze all the sequence of *Daldinia*. The RaxML 8.2.4 of CIPRES Science Gateway V.3.3 (https://www.phylo.org/) (accessed on 20 June 2024) was used for ML trees and bootstrap analyses (MLBS), and the GTR rate parameters were optimized with the BFGS method [22,23]. The AIC of Mr Modeltest 2.2 selected the best-fit model (GTR + I + G), MrBayes 3.0B4 computed branches’ Bayesian posterior probabilities (BPP), and hierarchical likelihood ratios (hLRTs) tested results [24,25]. Three million generations in four Markov chains were ran, sampled every one hundred generations, and a burn-in value to three thousand sampled trees was set. The PAUP*4.0b10 was used for the MP analysis. All characters used for analysis were weighted equally, and gaps were missing data [26]. When MLBS ≥ 70 or BPP ≥ 0.9, it can be considered as having a good support rate [7,19]. The phylogenetic trees were viewed and adjusted with FigTree v.1.4.4 (http://tree.bio.ed.ac.uk/software/figtree) (accessed on 20 June 2024) and beautified with Adobe Illustrator CC 2019.

## 3. Results

### 3.1. Phylogenetic Analyses

**Figure 1:**
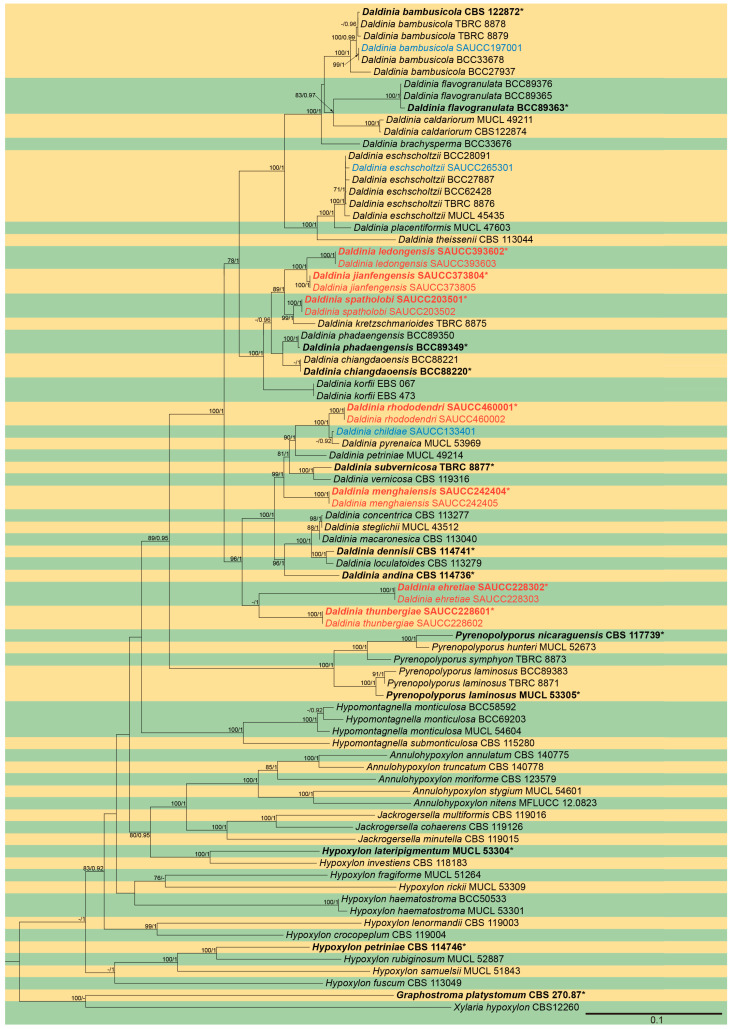
A maximum likelihood tree of Daldinia based on ITS, LSU, *rpb2*, and *tub2* gene sequences, and CBS 270.87 of *Graphostroma platystomum* and CBS 12260 of *Xylaria hypoxylon* as the tree root of *Daldinia*. The nodes of the branches are labeled MLBS/BPP (MLBS ≥ 70, BPP ≥ 0.9). The known species in this study are shown in blue font, while new species are shown in red font. Ex-type or ex-epitype strains are shown in bold with an “*”. Yellow and green parts are used to distinguish different strains. The black line at the bottom right is the scale bar, indicating 0.1 nucleotide changes at each site.

The phylogenetic analysis of ITS, LSU, *rpb2*, and *tub2* sequence data was performed to verify the interspecific relationships of *Daldinia*. A total of 84 sequences were used in this phylogenetic analysis, including 52 sequences of *Daldinia*, 12 sequences of *Hypoxylon*, 6 sequences of *Pyrenopolyporus*, 5 sequences of *Annulohypoxylon*, 4 sequences of *Hypomontagnella*, 3 sequences of *Jackrogersella*, and 1 sequence each of *Graphostroma* and *Xylaria*. A total of 6622 characters including gaps, 1714 of ITS, 2273 of LSU, 925 of *rpb2*, and 1710 of *tub2* were used in this phylogenetic analysis, including 4263 constant characters, 767 variable characters that are parsimony uninformative, and 1592 parsimony informative characters. The final ML Optimization Likelihood was −56,104.543344. The trees obtained through the employment of maximum likelihood (ML) and Bayesian algorithm (MB) methods exhibit a high degree of similarity. Figure 1 shows the ML tree with the most superior score, and the corresponding maximum likelihood bootstrap support values and Bayesian posterior probabilities (MLBS/BPP) were displayed above each of the branches. This study introduced seventeen strains of the genus *Daldinia* for phylogenetic analysis, which were divided into 10 clades on the phylogenetic tree, representing seven new species and three known species. It is worth noting that the sequences of a large number of strains discovered during this survey were the same, belonging to three different species. Therefore, to maintain the simplicity of the phylogenetic tree, *Daldinia bambusicola* (SAUCC197001), *D. childiae* (SAUCC133401), and *D. eschscholtzii* (SAUCC265301) were used as representative for phylogenetic analysis. Finally, 84 strains were divided into 56 taxa on the phylogenetic tree.

### 3.2. Sample Information Statistics

**Figure 2:**
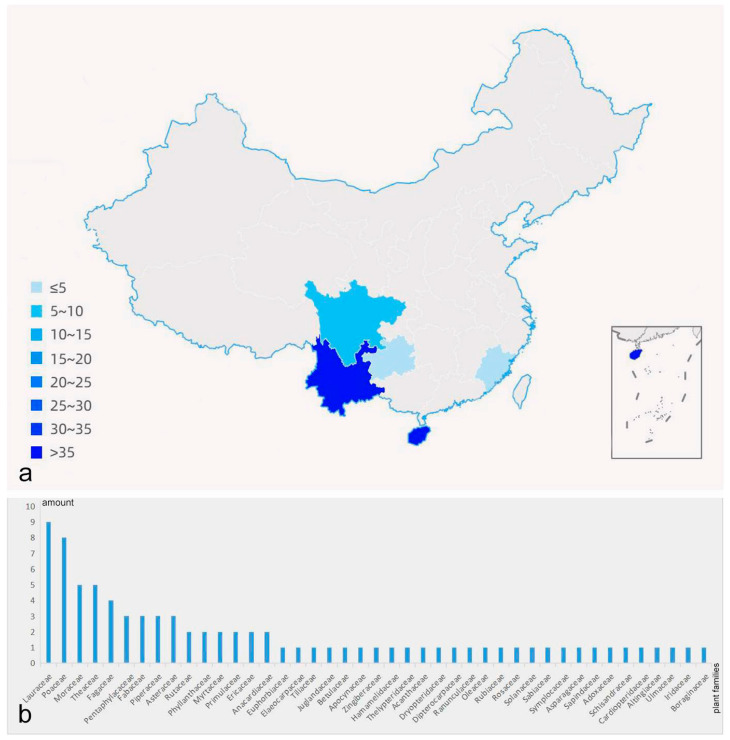
The distribution and host of *Daldinia*. (**a**) the number of *Daldinia* strains in five provinces of China; (**b**) the number of families to which the plant hosts of *Daldinia*.

In this investigation, a total of over 6000 samples were collected resulting in the isolation of *Daldinia* strains from 94 samples; we identified a total of three known species and seven new anamorphic species of the genus Daldinia; the collected information and host of each specimen are shown in Table 2, and the geographical distribution and host species are shown in Figure 2.

### 3.3. Taxonomy

**Figure 3:**
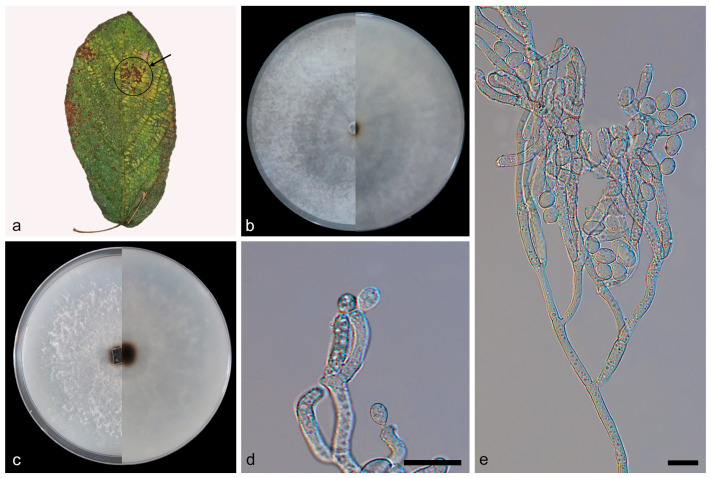
*Daldinia bambusicola* (type: SAUCC197001). (**a**) leaf of host *Viburnum rhytidophyllum*; (**b**) colony front and back after 7 days of culture on PDA; (**c**) colony front and back after 7 days of culture on OA; (**d**,**e**) conidiogenous cells and conidia. Scale bars: (**d**,**e**) 10 μm.

**Figure 4:**
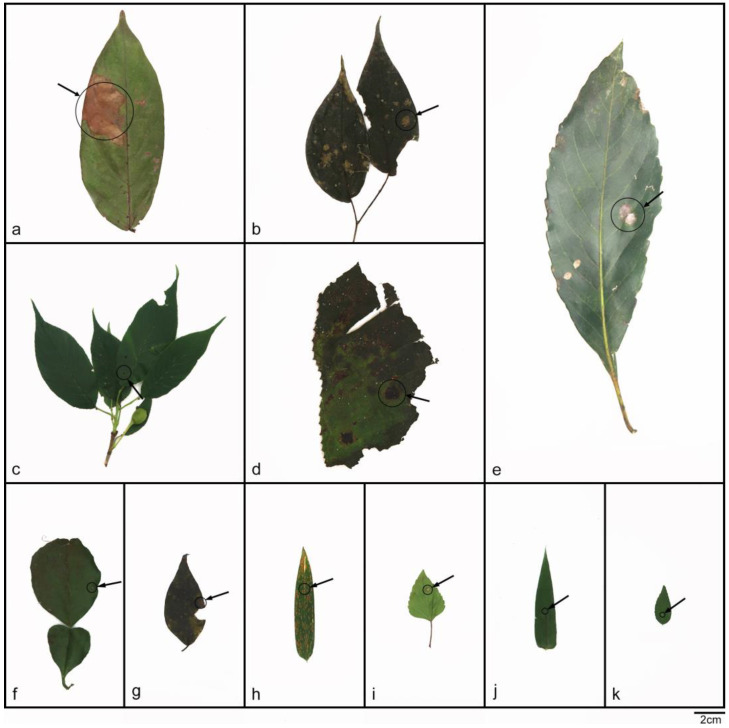
The host leaves of *Daldinia bambusicola*; relevant information is shown in Table 2. Scale bars: (**a**–**k**) 2 cm.

***Daldinia bambusicola* Y.M. Ju, J.D. Rogers and F. San Martín, Figure 3 and Figure 4.**

Description—conidiophores exhibit a virgariella-like to nodulisporium-like branching pattern [27]. Conidiophores dichotomously or trichotomously branched, finely roughened, hyaline, and aseptate, with 2–3 conidiogenous cells at each terminus, 110–160 × 2.1–2.7 µm (x = 132 × 2.5 µm, *n* = 22). Conidiogenous cells are cylindrical, finely roughened, and hyaline, with a flattened base, bearing conidia on their apical region, 10.1–15.3 × 2.5–3.1 μm ($\bar{x}$ = 12.2 × 2.7 µm, *n* = 24). Conidia are subglobose or ellipsoid, smooth or finely roughened, hyaline, aseptate, solitary, and produced holoblastically in sympodial sequence, 3.4–4.5 × 2.5–3.1 μm ($\bar{x}$ = 3.9 × 2.8 µm, *n* = 30). The teleomorph was not discovered.

Culture characteristics—after 7 days of cultivation on PDA medium at 25 °C, the colony exhibited a diameter of 90 mm and demonstrated a growth rate of approximately 12.9 mm/day. Colonies radiated and ringed from the middle to the periphery; flat, medium dense, and aerial mycelium was more on the periphery than in the center and was white (7443); the reverse center was black (419), and the periphery was white (7443). After 7 days of cultivation on OA medium at 25 °C, the colony exhibited a diameter of 75 mm and demonstrated a growth rate of approximately 10.7 mm/day. Colonies were rough, medium dense, and aerial mycelium distributed evenly in the middle and formed irregular continuous protrusions on the periphery and was white (7541); the reverse center was dark brown (1545), and the periphery was white (7443).

Notes—based on phylogenetic analysis and spore characteristics, the strains represented by SAUCC197001 were identified as *Daldinia bambusicola*, as shown in Figure 1 and Figure 3 [2,3]. These strains were distributed in the Yunnan, Hainan, Sichuan, and Guizhou provinces of China, especially in warm and humid forests and mountainous areas, which belong to a tropical monsoon climate and subtropical monsoon climate, and the altitude range was 151.57 m to 1876.71 m. These strains differ from those previously reported in that they were isolated from 12 new plant hosts (*Viburnum rhytidophyllum*, *Spatholobus suberectus*, *Piper nigrum*, *Cinnamomum verum*, *Koelreuteria paniculata*, *Ficus hirta*, *Schima superba*, *Citrus maxima*, *Phyllostachys heteroclada*, *Ageratina adenophora*, *Lophatherum gracile*, and *Ulmus pumila*), as shown in Figure 4.

**Figure 5:**
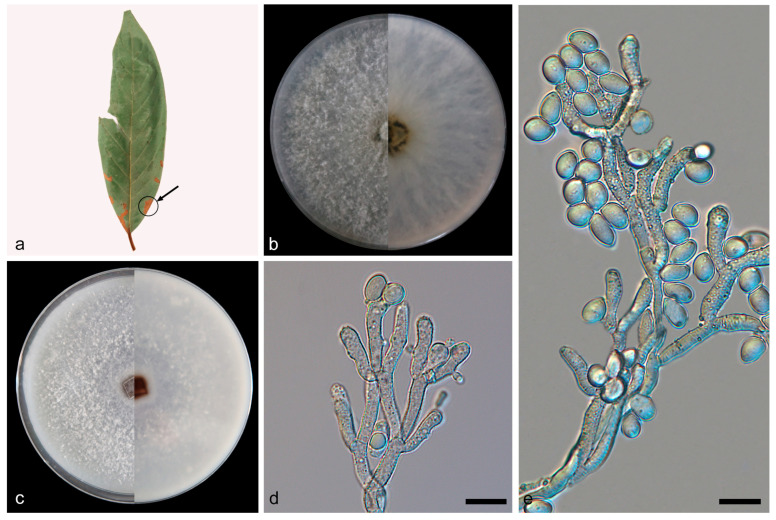
*Daldinia childiae* (type: SAUCC133401). (**a**) leaf of host *Machilus nanmu*; (**b**) colony front and back after 7 days of culture on PDA; (**c**) colony front and back after 7 days of culture on OA; (**d**,**e**) conidiogenous cells and conidia. Scale bars: (**d**,**e**) 10 μm.

**Figure 6:**
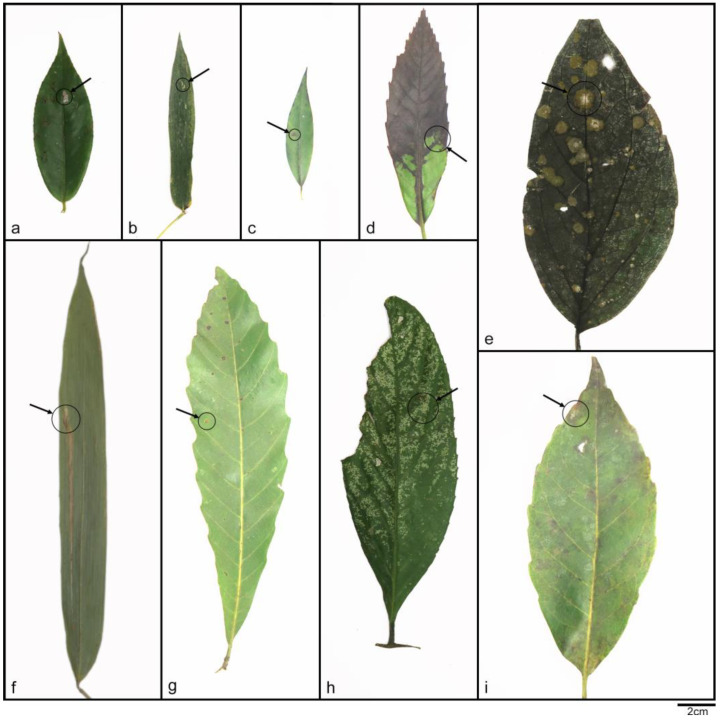
The host leaves of *Daldinia childiae*; relevant information is shown in Table 2. Scale bars: (**a**–**i**) 2 cm.

***Daldinia childiae* J.D. Rogers and Y.M. Ju, Figure 5 and Figure 6.**

Description—conidiophores exhibit a virgariella-like to nodulisporium-like branching pattern [27]. Conidiophores dichotomously or trichotomously branched and are smooth or finely roughened, hyaline, and aseptate, with 2–3 conidiogenous cells at each terminus, 150–220 × 2.5–3 µm ($\bar{x}$ = 181 × 2.7 µm, *n* = 20). Conidiogenous cells are clavate, terminal enlargement, roughened, and hyaline, bearing conidia on their apical region, 14.9–32.7 × 2.8–4.4 μm ($\bar{x}$ = 23.2 × 3.5 µm, *n* = 26). Conidia are subglobose or ellipsoid, smooth, pale yellow, hyaline, aseptate, solitary, and produced holoblastically in sympodial sequence, 5.8–9.1 × 4.1–5.8 μm ($\bar{x}$ = 7.6 × 4.9 µm, *n* = 30). The teleomorph was not discovered.

Culture characteristics—after 7 days of cultivation on PDA medium at 25 °C, the colony exhibited a diameter of 90 mm and demonstrated a growth rate of approximately 12.9 mm/day. Colonies were rough, dense, and aerial mycelium distributed evenly and was white (7443); the reverse center was black (419), light brown (1395) outwards, and the colony radiated from the middle to the periphery. After 7 days of cultivation on OA medium at 25 °C, the colony exhibited a diameter of 80 mm and demonstrated a growth rate of approximately 11.4 mm/day. Colonies were rough, dense, and aerial mycelium distributed evenly and formed many punctiform protrusions and was white (7541); the reverse center was brown (1615), and the periphery was white (7541).

Notes—J.D. Rogers et al. had reported *Daldinia childiae*, but it was not included in the phylogenetic analysis of *Daldinia* in recent publications [7,19,21,28]. In this paper, the complete sequence information of ITS, LSU, *rpb2*, and *tub2* of *D. childiae* was provided and included in the phylogenetic analysis. Based on sequence alignment and spore characteristics, the strains represented by SAUCC133401 were identified as *D. childiae*, as shown in Figure 1 and Figure 5 [3,28]. These strains were distributed in the Fujian, Yunnan, Hainan, and Sichuan provinces of China; their geographical distribution characteristics were similar to those of the *D. bambusicola* strains identified in this study. It is worth noting that Stadler et al. previously reported a Chinese specimen that resembles *D. childiae* but has an aberrant teleomorphic morphology [3]. The difference from previous reports is that the *D. childiae* found in this investigation was isolated from 10 new plant hosts (*Machilus nanmu*, *Eurya japonica*, *Piper nigrum*, *Microstegium vimineum*, *Pseudosasa japonica*, *Litsea cubeba*, *Quercus glauca*, *Schima superba*, *Castanopsis calathiformis*, and *Symplocos sumuntia*), as shown in Figure 6.

**Figure 7:**
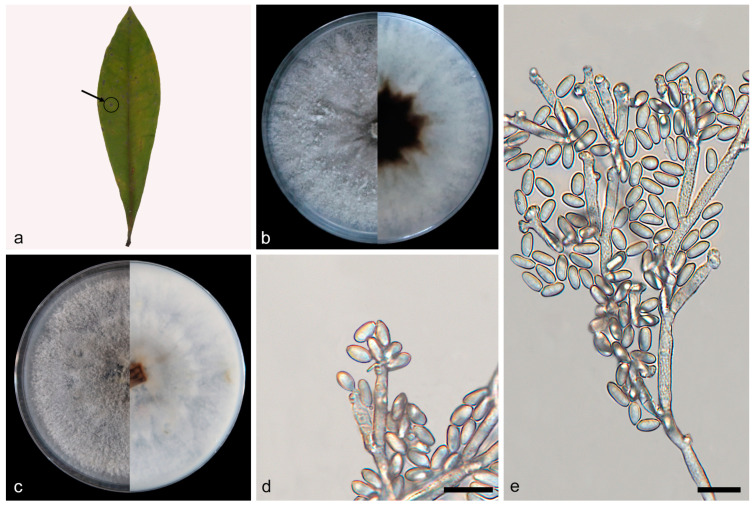
*Daldinia eschscholtzii* (type: SAUCC265301). (**a**) leaf of host *Lysimachia clethroides*; (**b**) colony front and back after 7 days of culture on PDA; (**c**) colony front and back after 7 days of culture on OA; (**d**,**e**) conidiogenous cells and conidia. Scale bars: (**d**,**e**) 10 μm.

**Figure 8:**
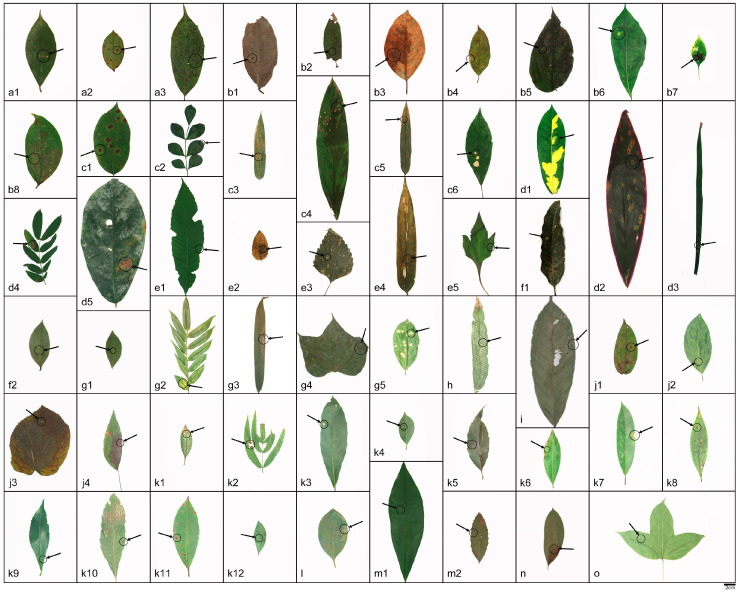
The host leaves of *Daldinia eschscholtzii*; relevant information is shown in Table 2. Scale bars: (**a1**–**o**) 2 cm.

***Daldinia eschscholtzii* Rehm, Figure 7 and Figure 8.**

Description—conidiophores exhibit a virgariella-like to nodulisporium-like branching pattern [27]. Conidiophores mononematous, dichotomously or trichotomously branched, finely roughened, hyaline, and aseptate, with 2–3 conidiogenous cells at each terminus, 120–214 × 2.3–4.1 µm ($\bar{x}$ = 176 × 3.4 µm, *n* = 21). Conidiogenous cells are cylindrical, finely roughened, and hyaline, with a flattened base, bearing conidia on their apical region, 14.9–22.7 × 2.1–3.6 μm ($\bar{x}$ = 18.3 × 2.7 µm, *n* = 26). Conidia are ellipsoid or dacryoid, smooth to finely roughened, hyaline, aseptate, and solitary, mostly with a flattened base and produced holoblastically in sympodial sequence, 4.9–6.8 × 2.3–3.5 μm ($\bar{x}$ = 5.7 × 2.8 µm, *n* = 30). The teleomorph was not discovered.

Culture characteristics—after 7 days of cultivation on PDA medium at 25 °C, the colony exhibited a diameter of 88 mm and demonstrated a growth rate of approximately 12.6 mm/day. Colonies radiated from the middle to the periphery, rough, medium dense, aerial mycelium was more on the periphery than in the center, and formed some protrusions and was white (7443); the reverse center formed a black (419) area with irregular edges surrounded by white (7443). After 7 days of cultivation on OA medium at 25 °C, the colony exhibited a diameter of 86 mm and demonstrated a growth rate of approximately 12.2 mm/day. Colonies were rough, medium dense, and aerial mycelium distributed evenly and was white (7443); the reverse center was light brown (1395), the periphery was white (7443), and multiple rings appeared from the middle to the periphery.

Notes—based on phylogenetic analysis and spore characteristics, the strains represented by SAUCC265301 were identified as *Daldinia eschscholtzii*, as shown in Figure 1 and Figure 7 [3]. According to the survey, it was extensively found in the Fujian, Yunnan, Hainan, Sichuan, and Guizhou provinces of China. Particularly prevalent in warm, moist and vegetated forests, mountains, plains, and wetlands at elevations ranging from 154.85 m to 1876.7 m. This survey has discovered 58 new plant hosts of *Daldinia*; they belong to 38 families, mainly *Lauraceae*, *Moraceae*, and *Poaceae*, as shown in Figure 8.

**Figure 9:**
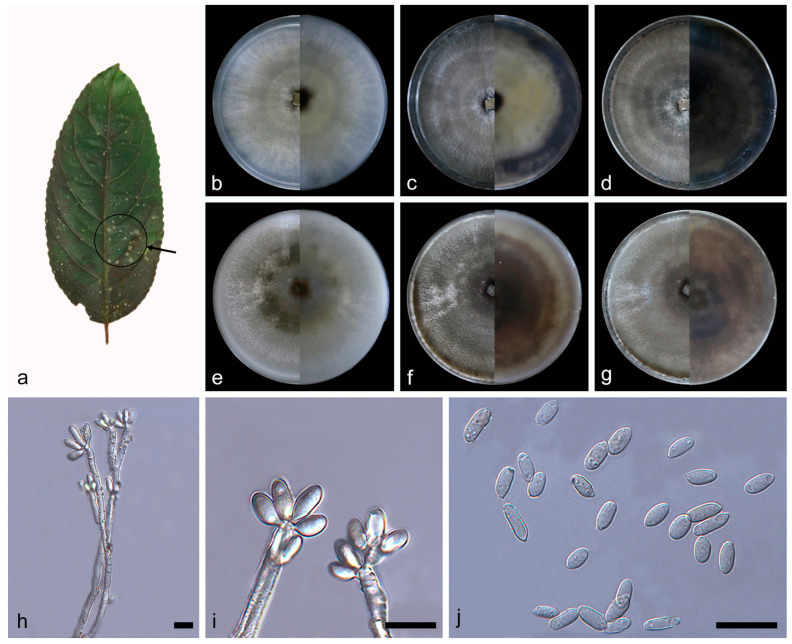
*Daldinia ehretiae* (holotype: HMAS352914). (**a**) leaf of host plant *Ehretia acuminata*; (**b**–**d**) colony front and back after 7 days, 14 days, and 31 days culture on PDA; (**e**–**g**) colony front and back after 7 days, 14 days, and 31 days culture on OA; (**h**–**j**) conidiogenous cells and conidia. Scale bars: (**h**–**j**) 10 μm.

***Daldinia ehretiae* C.Z. Yin, Z.X. Zhang and X.G. Zhang, sp. nov. Figure 9.**

MycoBank—MB853131

Etymology—the epithet “*ehretiae*” refers to the name of the host plant, *Ehretia acuminata*.

Material studied—China, Yunnan Province, Jinghong City, Sancha River, 22°10′10″ N, 100°51′49″ E, on diseased leaves of *Ehretia acuminata*, 19 March 2023, C.Z. Yin, Z.X. Zhang and X.G. Zhang, holotype HMAS352914, ex-type culture SAUCC228302.

Description—conidiophores exhibit a virgariella-like to nodulisporium-like branching pattern [27]. Conidiophores mononematous or dichotomously branched and are finely roughened, hyaline, and aseptate, with 1–2 conidiogenous cells at each terminus, 100–210 × 3.1–4.3 µm ($\bar{x}$ = 165 × 3.6 µm, *n* = 23). Conidiogenous cells are cylindrical, finely roughened, and hyaline, bearing conidia on their apical region, 16.8–24.5 × 2.7–4.1 µm ($\bar{x}$ = 19.3 × 3.5 µm, *n* = 25). Conidia are ellipsoid or cylindrical, smooth to finely roughened, hyaline, aseptate, and solitary, mostly with a flattened base, and produced holoblastically in sympodial sequence, 4.2–6.6 × 1.7–2.8 µm ($\bar{x}$ = 5.4 × 2.2 µm, *n* = 30). The teleomorph was not discovered.

Culture characteristics—after 7 days of cultivation on PDA medium at 25 °C, colonies radiated and ringed from the middle to the periphery, showing black (419), pale green (365), grey (422), and white (7541) in sequence, flat, medium dense, and aerial mycelium distributed evenly; the reverse was similar to the front. After 14 days, colonies formed multiple white (7443) rings, dense, and aerial mycelium grey (423); the reverse center and periphery was black (426), and the intermediate part was light green (373). After 31 days, colonies changed from gray to turquoise brown (403); the reverse was mostly black (426). The average growth rate on PDA medium was 11.7 mm/day. After 7 days of cultivation on OA medium at 25 °C, colonies formed a dark green (378) ring, and the rest was mostly white (7443), rough, medium dense, and aerial mycelium was more on the periphery than in the center; the reverse was similar to the front. After 14 days, colonies alternated gray (425) and white (7541) rings from the middle to the periphery, dense, and aerial mycelium distributed evenly; the reverse alternated brown (1615) and white (7443) rings from the middle to the periphery. After 31 days, colonies changed from gray to light gray (427); the reverse was mostly brown (1615). The average growth rate on OA medium was 11.6 mm/day.

Additional material studied—China, Yunnan Province, Jinghong City, Sancha River, 22°10′10″ N, 100°51′49″ E, on diseased leaves of *Ehretia acuminata*, 19 March 2023, C.Z. Yin, Z.X. Zhang and X.G. Zhang, HSAUP 228303, living culture SAUCC228303.

Notes—the phylogenetic analysis of ITS, LSU, *rpb2*, and *tub2* sequences revealed a close relationship between *Daldinia ehretiae* and another newly discovered species, *D. thunbergiae*. *Daldinia ehretiae* is different from *D. thunbergiae* by 69/559 bp in ITS, 17/1259 bp in LSU, and 201/1393 bp in *tub2*. Morphologically, *D. ehretiae* and *D. thunbergiae* differ in that there are a fewer number of conidiogenous cells (1–2 × 1–3) at the terminus of conidiophores and larger conidiogenous cells (16.8–24.5 × 2.7–4.1 vs. 6.7–17.3 × 1.9–2.5 µm). In addition, there is little difference in conidia size between *D. ehretiae* and *D. thunbergiae*, but the conidial shape of *D. ehretiae* is mostly ellipsoid or cylindrical, and the conidia of *D. thunbergiae* are mostly teardrop shaped except ellipsoid. Therefore, *D. ehretiae* can be identified as a new anamorphic species of *Daldinia* via phylogenetic and morphological comparison.

**Figure 10:**
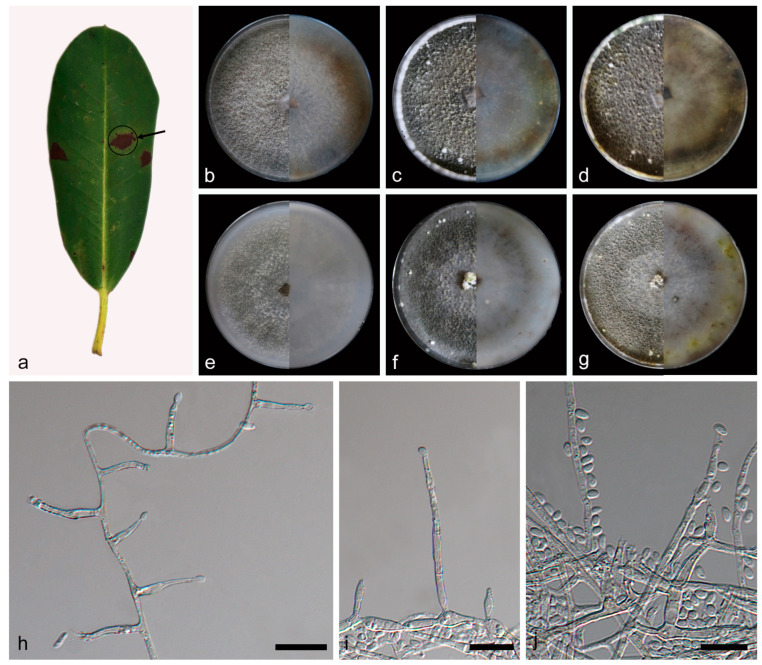
*Daldinia rhododendri* (holotype: HMAS352918). (**a**) leaf of host plant *Rhododen dron decorum*; (**b**–**d**) colony front and back after 7 days, 14 days, and 31 days culture on PDA; (**e**–**g**) colony front and back after 7 days, 14 days, and 31 days culture on OA; (**h**–**j**) conidiogenous cells and conidia. Scale bars: (**h**–**j**) 10 μm.

***Daldinia rhododendri* C.Z. Yin, Z.X. Zhang and X.G. Zhang, sp. nov. Figure 10.**

MycoBank—MB853135

Etymology—the epithet “*rhododendri*” refers to the name of the host plant, *Rhododendron decorum*.

Material studied—China, Yunnan Province, Diqing Prefecture, Shangri-la City, 27°58′43″ N, 99°34′24″ E, on diseased leaves of *Rhododendron decorum*, 28 June 2023, C.Z. Yin, Z.X. Zhang and X.G. Zhang, holotype HMAS352918, ex-type culture SAUCC460001.

Description—conidiophores exhibit a virgariella-like to nodulisporium-like branching pattern [27]. Conidiophores mononematous or dichotomously branched and are rare, smooth or finely roughened, hyaline, and septate, with conidiogenous cells at each terminus, 40–90 × 1.4–2.0 µm ($\bar{x}$ = 74 × 1.7 µm, *n* = 21). Conidiogenous cells are cylindrical or ampuliform, rare, smooth to finely roughened, and hyaline, bearing conidia on their apical region, 5.9–11.6 × 1.1–2.9 µm ($\bar{x}$ = 8.6 × 2.3 µm, *n* = 26). Conidia are ellipsoid, cylindrical or banana shaped, smooth, hyaline, aseptate, solitary, and produced from percurrently proliferating conidiogenous cells, 3.2–5.1 × 1.1–2.3 µm ($\bar{x}$ = 4.1 × 1.7 µm, *n* = 30). The teleomorph was not discovered.

Culture characteristics—after 7 days of cultivation on PDA medium at 25 °C, colonies were rough, dense, and aerial mycelium distributed evenly and was white (7443) and grey (424) from the middle out; the reverse center was white (7443), with irregular areas of reddish brown (469) around. After 14 days, colonies formed a few white (7541) fluffy balls and a circle of white (7541) fluffy mycelium around; aerial mycelium was dark grey (425); the reverse center was white (7443), with irregular areas of reddish brown (469) and pale green (384) around. After 31 days, colonies had not changed significantly; the reverse was predominantly brown (1405) and black (426). The average growth rate on PDA medium was 12 mm/day. After 7 days of cultivation on OA medium at 25 °C, colonies alternated dark gray (425) and white (7443) with rings from the middle to the periphery and was rough, medium dense, with aerial mycelium distributed evenly; the reverse was white (7443). After 14 days, colonies formed a few white (7541) fluffy balls, dense; the reverse exhibited an irregular dark gray (425) ring. After 31 days, colonies changed from dark gray to gray (423); the reverse exhibited some irregular yellow green (582) areas. The average growth rate on OA medium was 11.1 mm/day.

Additional material studied—China, Yunnan Province, Diqing Prefecture, Shangri-la City, 27°58′43″ N, 99°34′24″ E, on diseased leaves of *Rhododendron decorum*, 28 June 2023, C.Z. Yin, Z.X. Zhang and X.G. Zhang, HSAUP 460002, living culture SAUCC460002.

Notes—the phylogenetic analysis of ITS, LSU, *rpb2*, and *tub2* sequences revealed a close relationship between *Daldinia rhododendri*, *D. pyrenaica*, and *D. childiae*, and they are both on a branch with better support (MLBS/BPP = 100/1). *Daldinia rhododendri* is different from *D. pyrenaica* by 21/552 bp in ITS, 4/982 bp in LSU, and 29/1342 bp in *tub2* and different from *D. childiae* by 20/552 bp in ITS, 6/982 bp in LSU, and 18/1368 bp in *tub2*. Morphologically, *D. rhododendri* and *D. pyrenaica* differ obviously in conidiogenous cells (5.9–11.6 × 1.1–2.9 vs. 10.0–25.0 × 2.5–3.0 µm) and conidia (3.2–5.1 × 1.1–2.3 vs. 6.5–7.0 × 4.0–5.0 μm) [3,29]. *Daldinia rhododendri* and *D. childiae* also differ obviously in conidiogenous cells (5.9–11.6 × 1.1–2.9 vs. 10.0–25.0 × 3.0–4.0 µm) and conidia (3.2–5.1 × 1.1–2.3 vs. 7.0–9.0 × 4.5–5.5 μm) [3,28]. Therefore, *D. rhododendri* can be identified as a new anamorphic species of *Daldinia* by phylogenetic and morphological comparison.

**Figure 11:**
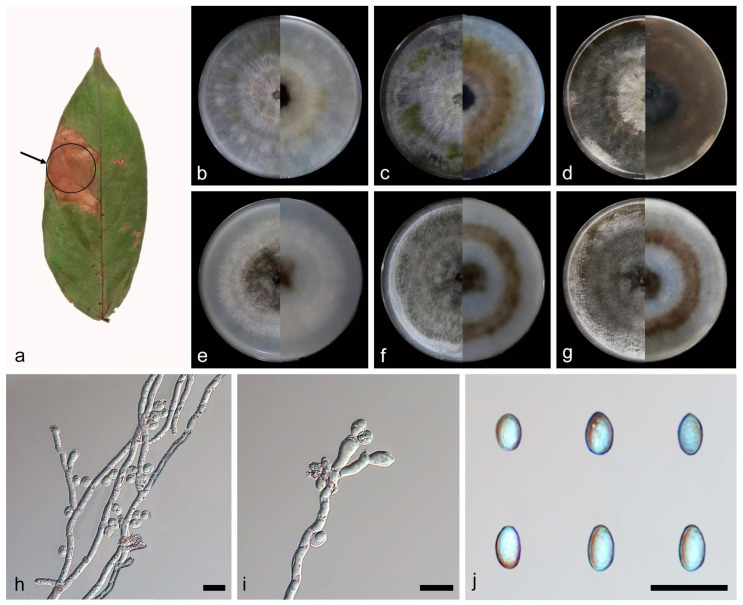
*Daldinia spatholobi* (holotype: HMAS352919). (**a**) leaf of host plant *Spatholobus suberectus*; (**b**–**d**) colony front and back after 7 days, 14 days, and 31 days culture on PDA; (**e**–**g**) colony front and back after 7 days, 14 days, and 31 days culture on OA; (**h**–**j**) conidiogenous cells and conidia. Scale bars: (**h**–**j**) 10 μm.

***Daldinia spatholobi* C.Z. Yin, Z.X. Zhang and X.G. Zhang, sp. nov. Figure 11.**

MycoBank—MB853136

Etymology—the epithet “*spatholobi*” refers to the name of the host plant, *Spatholobus suberectus*.

Material studied—China, Yunnan Province, Jinghong City, Xishuangbanna primitive Forest Park, 22°1′52″ N, 100°52′36″ E, on diseased leaves of *Spatholobus suberectus*, 17 March 2023, C.Z. Yin, Z.X. Zhang and X.G. Zhang, holotype HMAS352919, ex-type culture SAUCC203501.

Description—Conidiophores exhibit a virgariella-like to nodulisporium-like branching pattern [27]. Conidiophores mononematous, dichotomously, or trichotomously branched and are roughened, hyaline, and septate, with 1–3 conidiogenous cells at each terminus, 90–150 × 2.9–4.1 µm ($\bar{x}$ = 135 × 3.4 µm, *n* = 22). Conidiogenous cells are cylindrical or clavate, roughened, and hyaline, with a flattened base, bearing conidia on their apical region, 9.5–14.9 × 2.4–3.0 µm ($\bar{x}$ = 12.3 × 2.6 µm, *n* = 25). Conidia are ellipsoid or teardrop shaped, smooth to finely roughened, hyaline, aseptate, solitary, and produced holoblastically in sympodial sequence, 4.3–6.1 × 2.6–3.7 µm ($\bar{x}$ = 5.6 × 3.4 µm, *n* = 30). The teleomorph was not discovered.

Culture characteristics—after 7 days of cultivation on PDA medium at 25 °C, colonies alternated pale brown (1245), white (7443), pale green (390), and white (7443) with rings from the middle to the periphery and were flat, medium dense, and with aerial mycelium more in the center than on the periphery; the reverse was similar to the front, but the center was black (419). After 14 days, colonies formed irregular green (377) areas and was dense with aerial mycelium distributed evenly; the reverse center was black (419), brown (1255) with irregular area outside, and white (7443) outwards. After 31 days, colonies were white (7527) in the center, and the rest was mostly black (419); the reverse was also mostly brown (1405). The average growth rate on PDA medium was 10.6 mm/day. After 7 days of cultivation on OA medium at 25 °C, colonies formed a black (426) circular area in the center and was white (7443) outwards and flat, medium dense, and with aerial mycelium distributed evenly; the reverse center was brown (1405) and mostly white (7443) on the outside. After 14 days, colonies ringed from the middle to the periphery, showing black (426), pale brown (1395), grey (423), dark green (371), and white (7541) colors and were dense; the reverse center was black (419) and formed a regular brown (1405) ring. After 31 days, colonies darkened in color; the reverse has not changed significantly. The average growth rate on OA medium was 9.7 mm/day.

Additional material studied—China, Yunnan Province, Jinghong City, Xishuangbanna primitive Forest Park, 22°1′52″ N, 100°52′36″ E, on diseased leaves of *Spatholobus suberectus*, 17 March 2023, C.Z. Yin, Z.X. Zhang and X.G. Zhang, HSAUP 203502, living culture SAUCC203502.

Notes—the phylogenetic analysis of ITS, LSU, *rpb2*, and *tub2* sequences revealed a close relationship between *Daldinia spatholobi* and *D. kretzschmarioides*, and they are both on a branch with better support (MLBS/BPP = 99/1). *Daldinia spatholobi* was different from *D. kretzschmarioides* by 6/961 bp in LSU and 25/1066 bp in *tub2*. Morphologically, compared to *D. kretzschmarioides*, *D. spatholobi* has shorter conidiogenous cells (2.4–3.0 vs. 3.0–4.0 µm) and similar conidia sizes. But the conidia of *D. spatholobi* are teardrop shaped in addition to ellipsoid, with a finely roughened surface. Under the same conditions, *D. spatholobi* exhibits regular circular pigment precipitation on OA medium with fewer mycelium, while *D. kretzschmarioides* exhibits irregular pigment precipitation with more mycelium [7]. Therefore, *D. spatholobi* can be identified as a new anamorphic species of *Daldinia* by phylogenetic and morphological comparison.

**Figure 12:**
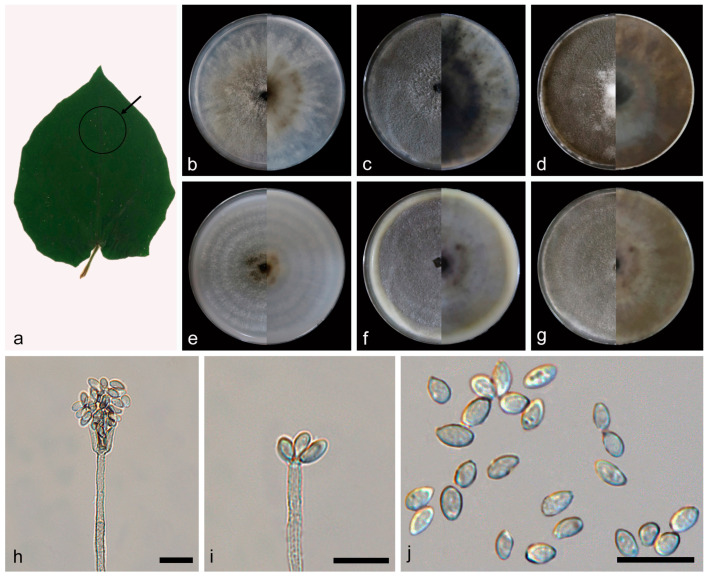
*Daldinia thunbergiae* (holotype: HMAS352920). (**a**) leaf of host plant *Thunbergia grandiflora*; (**b**–**d**) colony front and back after 7 days, 14 days, and 31 days culture on PDA; (**e**–**g**) colony front and back after 7 days, 14 days, and 31 days culture on OA; (**h**–**j**) conidiogenous cells and conidia. Scale bars: (**h**–**j**) 10 μm.

***Daldinia thunbergiae* C.Z. Yin, Z.X. Zhang and X.G. Zhang, sp. nov. Figure 12.**

MycoBank—MB853137

Etymology—the epithet “*thunbergiae*” refers to the name of the host plant, *Thunbergia grandiflora*.

Material studied—China, Yunnan Province, Jinghong City, Sancha River, 22°10′10″ N, 100°51′49″ E, on diseased leaves of *Thunbergia grandiflora*, 19 March 2023, C.Z. Yin, Z.X. Zhang and X.G. Zhang, holotype HMAS352920, ex-type culture SAUCC228601.

Description—Conidiophores exhibit a virgariella-like to nodulisporium-like branching pattern [27]. Conidiophores mononematous, dichotomously, or trichotomously branched and are roughened, hyaline, and septate, with 1–4 conidiogenous cells at each terminus, 70–220 × 1.8–4.1 µm ($\bar{x}$ = 150 × 3.3 µm, *n* = 21). Conidiogenous cells are cylindrical or clavate, finely roughened, and hyaline, bearing conidia on their apical region, 6.7–17.3 × 1.9–2.5 µm ($\bar{x}$ = 11.5 × 2.2 µm, *n* = 26). Conidia are ellipsoid or teardrop shaped, finely roughened, hyaline, aseptate, solitary, mostly with a flattened base, and produced holoblastically in sympodial sequence, 3.2–5.0 × 2.2–3.1 µm ($\bar{x}$ = 4.2 × 2.7 µm, *n* = 30). The teleomorph was not discovered.

Culture characteristics—after 7 days of cultivation on PDA medium at 25 °C, colonies radiated from the middle to the periphery, showing white (7443), dark green (385), and white (7443) colors and were rough, medium dense, and with aerial mycelium more in the center than on the periphery; the reverse center was dark green (385), with an incomplete dark green (385) ring outwards, and the rest was white (7443). After 14 days, colonies were mostly dark gray (425), plat, dense, and with aerial mycelium distributed evenly; the reverse center was mostly black (426), surrounded by many black (426) spots and irregular pale green (391) areas, and the rest was white (7443). After 31 days, colonies changed from dark gray to dark brown (1545); white (7541) mycelium appeared in the center; the reverse periphery was mostly brown (1615). The average growth rate on PDA medium was 11.1 mm/day. After 7 days of cultivation on OA medium at 25 °C, colonies formed multiple white (7541) rings and the center was black (419), plat, and medium dense, with aerial mycelium distributed evenly; the reverse was similar to the front, but the center was brown (1615). After 14 days, colonies formed a white (7443) ring in the outermost ring and the rest was mostly gray (424) and dense; the reverse was similar to the front, but the center and some irregular parts were dark green (378). After 31 days, colonies changed from gray to light brown (1255); the reverse was also mostly brown (1265). The average growth rate on OA medium was 11.3 mm/day.

Additional material studied—China, Yunnan Province, Jinghong City, Sancha River, 22°10′10″ N, 100°51′49″ E, on diseased leaves of *Thunbergia grandiflora*, 19 March 2023, C.Z. Yin, Z.X. Zhang and X.G. Zhang, HSAUP 228602, living culture SAUCC228602.

Notes—*Daldinia thunbergiae* is closely related to another newly discovered species in this study, *D. ehretiae*, on the phylogenetic tree of *Daldinia*. Based on the differences in sequence, conidiophores, conidiogenous cells, and conidia between the two species mentioned above, *D. thunbergiae* can be identified as a new anamorphic species of *Daldinia*.

**Figure 13:**
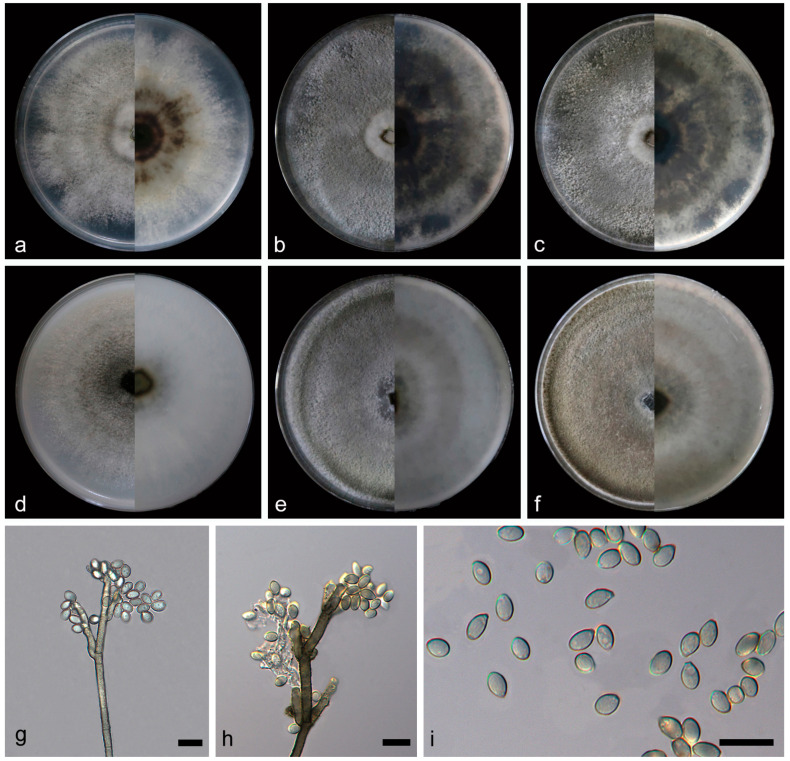
*Daldinia jianfengensis* (holotype: HMAS352915). (**a**–**c**) colony front and back after 7 days, 14 days, and 31 days culture on PDA; (**d**–**f**) colony front and back after 7 days, 14 days, and 31 days culture on OA; (**g**–**i**) conidiogenous cells and conidia. Scale bars: (**g**–**i**) 10 μm.

***Daldinia jianfengensis* C.Z. Yin, Z.X. Zhang and X.G. Zhang, sp. nov. Figure 13.**

MycoBank—MB853132

Etymology—the epithet “*jianfengensis*” refers to the name of the strain location, Jianfeng Town.

Material studied—China, Hainan Province, Ledong County, Jianfeng Town, 18°41′19″ N, 108°51′31″ E, on decayed leaves, 12 April 2023, C.Z. Yin, Z.X. Zhang and X.G. Zhang, holotype HMAS352915, ex-type culture SAUCC373804.

Description—conidiophores exhibit a virgariella-like to nodulisporium-like branching pattern [27]. Conidiophores mononematous, dichotomously, or trichotomously branched and are finely roughened, hyaline, and septate, with 1–4 conidiogenous cells at each terminus, 70–120 × 2.9–4.4 µm ($\bar{x}$ = 97 × 3.6 µm, *n* = 22). Conidiogenous cells are cylindrical, finely roughened, and hyaline, bearing conidia on their apical region, 12.1–16.9 × 2.6–3.6 µm ($\bar{x}$ = 15.1 × 3.2 µm, *n* = 25). Conidia are subglobose or ellipsoid, smooth to finely roughened, hyaline, aseptate, solitary, mostly with a flattened base, and produced holoblastically in sympodial sequence, 3.2–5.5 × 2.6–3.7 µm ($\bar{x}$ = 4.3 × 3.5 µm, *n* = 30). The teleomorph was not discovered.

Culture characteristics—after 7 days of cultivation on PDA medium at 25 °C, colonies exhibited alternated white (7443) and gray (422) rings from the middle to the periphery, with periphery irregularity, flat, and medium dense; the reverse center was black (426), dark green (385) irregular area outside, and white (7443) outwards. After 14 days, colonies formed a round, white (7443) fluff-like area in the center, dense, and aerial mycelium distributed evenly, grey (424); the reverse exhibited alternating black (419) and white (7443) irregular rings from the middle to the periphery. After 31 days, colonies and reverse had not changed significantly. The average growth rate on PDA medium was 11.6 mm/day. After 7 days of cultivation on OA medium at 25 °C, colonies were black (419), gray (425), and white (7443) from the middle out, flat, and medium dense; the reverse center was gray (424), with a circle of black (419) on the outside and mostly white (7541) on the outside. After 14 days, colonies alternated gray (424) and white (7443) from the middle to the periphery, dense, and aerial mycelium distributed evenly; the reverse alternated gray (423) and white (7443) with regular rings from the middle to the periphery. After 31 days, colonies changed from gray to pale brown (1265); the reverse was slightly darker. The average growth rate on OA medium was 11.4 mm/day.

Additional material studied—China, Hainan Province, Ledong County, Jianfeng Town, 18°41′19″ N, 108°51′31″ E, on decayed leaves, 12 April 2023, C.Z. Yin, Z.X. Zhang and X.G. Zhang, HSAUP 373805, living culture SAUCC373805.

Notes—The phylogenetic analysis of ITS, LSU, *rpb2*, and *tub2* sequences revealed a close relationship between *Daldinia jianfengensis* and another newly discovered species, *D. ledongensis*, and they are both on a branch with better support (MLBS/BPP = 100/1). *D. jianfengensis* was different from *D. ledongensis* by 56/549 bp in ITS, 12/1277 bp in LSU, and 5/1038 bp in *tub2*. Morphologically, the difference between *D. jianfengensis* and *D. ledongensis* is that there are more conidiogenous cells (1–3 × 1–2) at the terminus of conidiophores and conidiogenous cells are generally long (12.1–16.9 vs. 8.6–15.1 µm) and are mostly distributed at the terminus of conidiophores. In addition, the conidia of *D. jianfengensis* are wider (2.6–3.7 vs. 1.4–2.0 μm) and produce more conidia under the same conditions. The conidial shape of *D. jianfengensis* is subglobose or ellipsoid, which is different from that of the conidia of *D. ledongensis* with an ellipsoid or fusiform shape. It is worth noting that under the same culture conditions, whether in OA medium or PDA medium, the medium of *D. jianfengensis* will show circular dark pigmentation, and the mycelium will be darker, while the medium of *D. ledongensis* only has a small part of pigmentation and the mycelium was always white. Therefore, *D. jianfengens* can be identified as a new anamorphic species of *Daldinia* by phylogenetic and morphological comparison.

**Figure 14:**
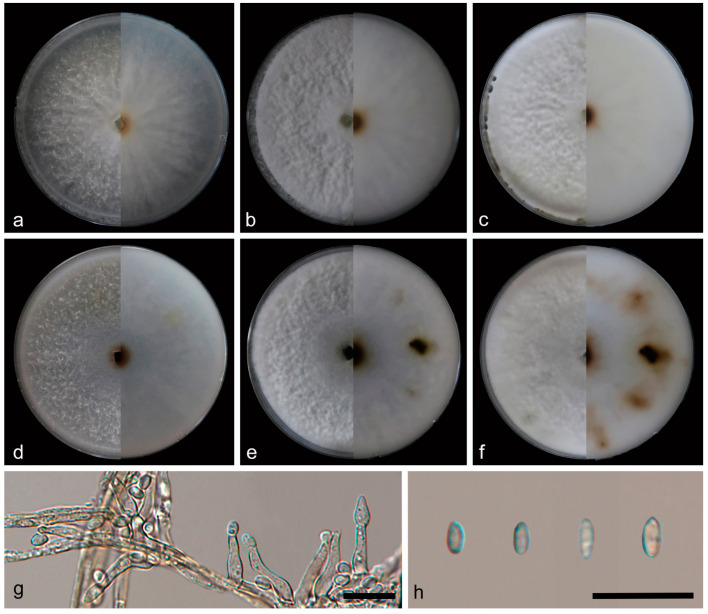
*Daldinia ledongensis* (holotype: HMAS352916). (**a**–**c**) colony front and back after 7 days, 14 days, and 31 days culture on PDA; (**d**–**f**) colony front and back after 7 days, 14 days, and 31 days culture on OA; (**g**,**h**) conidiogenous cells and conidia. Scale bars: (**g**,**h**) 10 μm.

***Daldinia ledongensis* C.Z. Yin, Z.X. Zhang and X.G. Zhang, sp. nov. Figure 14.**

MycoBank—MB853133

Etymology—the epithet “*ledongensis*” refers to the name of the strain location, Ledong County.

Material studied—China, Hainan Province, Ledong County, 18°41′18″ N, 108°51′31″ E, on decayed leaves, 12 April 2023, C.Z. Yin, Z.X. Zhang and X.G. Zhang, holotype HMAS352916, ex-type culture SAUCC393602.

Description—Conidiophores exhibit a virgariella-like to nodulisporium-like branching pattern [27]. Conidiophores mononematous or dichotomously branched and are rare, smooth or finely roughened, hyaline, and aseptate, with a conidiogenous cells at each terminus, 120–200 × 1.7–2.1 µm ($\bar{x}$ = 157 × 1.8 µm, *n* = 21). Conidiogenous cells are clavate, rare, smooth to finely roughened, and hyaline, with a flattened base, bearing conidia on their apical region, 8.6–15.1 × 1.2–3.4 µm ($\bar{x}$ = 11.3 × 2.4 µm, *n* = 24). Conidia are ellipsoid or fusiform, smooth to finely roughened, hyaline, aseptate, solitary, and produced from percurrently proliferating conidiogenous cells, 3.2–4.0 × 1.4–2.0 µm ($\bar{x}$ = 3.5 × 1.6 µm, *n* = 30). The teleomorph was not discovered.

Culture characteristics—after 7 days of cultivation on PDA medium at 25 °C, colonies radiated from the middle to the periphery, rough, medium dense, with aerial mycelium more on the periphery than in the center and was white (7443); the reverse was similar to the front, but the center was hazel (471). After 14 days, colonies were dense, and aerial mycelium distributed evenly; the reverse was similar to the front, but the center was brown (1405). After 31 days, colonies became denser; the reverse had not changed significantly. The average growth rate on PDA medium was 10.7 mm/day. After 7 days of cultivation on OA medium at 25 °C, colonies were rough, medium dense, and aerial mycelium was more on the periphery than in the center and was white (7443); the reverse center was brown (1615), mostly white (7443) on the outside. After 14 days, colonies were dense; the reverse exhibited some dark brown (1545) areas. After 31 days, colonies became denser; the reverse exhibited many irregular brown (1395) areas. The average growth rate on OA medium was 11.4 mm/day.

Additional material studied—China, Hainan Province, Ledong County, 18°41′18″ N, 108°51′31″ E, on decayed leaves, 12 April 2023, C.Z. Yin, Z.X. Zhang and X.G. Zhang, HSAUP 393603, living culture SAUCC393603.

Notes—*Daldinia ledongensis* was closely related to another newly discovered species, *D. jianfengensis*, on the phylogenetic tree of *Daldinia*. Based on the differences in sequence, conidiophores, conidiogenous cells, conidia, and medium between the two species mentioned above, *D. ledongensis* can be identified as a new anamorphic species of *Daldinia*.

**Figure 15:**
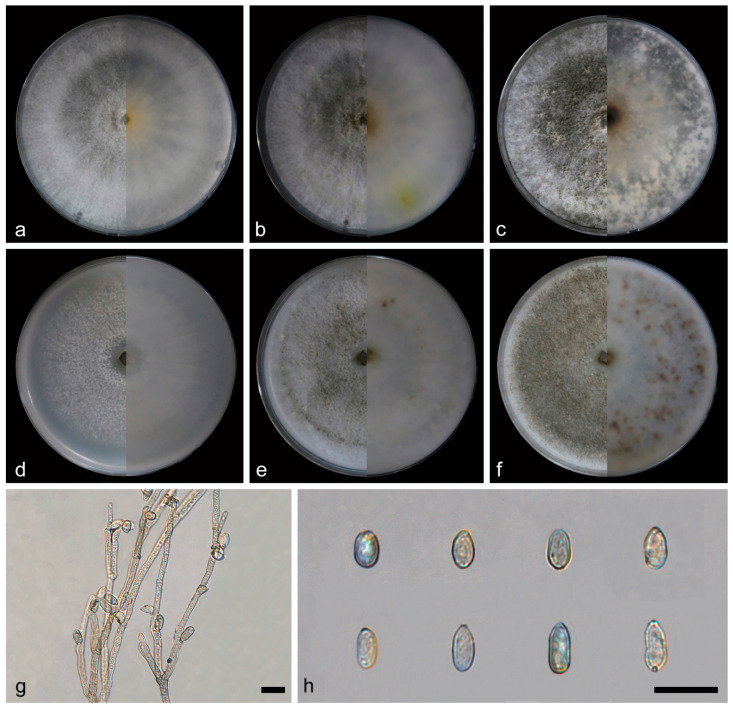
*Daldinia menghaiensis* (holotype: HMAS352917). (**a**–**c**) colony front and back after 7 days, 14 days, and 31 days culture on PDA; (**d**–**f**) colony front and back after 7 days, 14 days, and 31 days culture on OA; (**g**,**h**) conidiogenous cells and conidia. Scale bars: (**g**,**h**) 10 μm.

***Daldinia menghaiensis* C.Z. Yin, Z.X. Zhang and X.G. Zhang, sp. nov. Figure 15.**

MycoBank—MB853134

Etymology—the epithet “*menghaiensis*” refers to the name of the strain location, Menghai County.

Material studied—China, Yunnan Province, Menghai County, 21°55′25″ N, 100°35′41″ E, on decayed leaves, 18 March 2023, C.Z. Yin, Z.X. Zhang and X.G. Zhang, holotype HMAS352917, ex-type culture SAUCC242404.

Description—Conidiophores exhibit a virgariella-like to nodulisporium-like branching pattern [27]. Conidiophores dichotomously or trichotomously branched, occasionally branched from the conidiogenous region, and are finely roughened, hyaline, and septate, with 1–2 conidiogenous cells at each terminus, 80–150 × 1.9–3.4 µm ($\bar{x}$ = 115 × 2.6 µm, *n* = 22). Conidiogenous cells are cylindrical or clavate, finely roughened, and hyaline, bearing conidia on their apical region, 16.9–23.5 × 2.0–3.5 µm ($\bar{x}$ = 19.3 × 2.8 µm, *n* = 27). Conidia are ellipsoid, subglobose or dacryoid, roughened, hyaline, aseptate, solitary, mostly with flattened base, and produced holoblastically in sympodial sequence, 4.7–8.2 × 3.1–4.0 µm ($\bar{x}$ = 5.7 × 3.5 µm, *n* = 30). The teleomorph was not discovered.

Culture characteristics—after 7 days of cultivation on PDA medium at 25 °C, colonies radiated and ringed from the middle to the periphery, flat, medium dense, and with aerial mycelium more on the periphery than in the center and was white (7443); the reverse was similar to the front, but the center was pale brown (1375). After 14 days, colonies were dense; the reverse center was brown (1395), there appeared some light green (382) areas. After 31 days, colonies became rough; the reverse center was black (419), and the shape of the colonies became irregular. The average growth rate on PDA medium was 12.8 mm/day. After 7 days of cultivation on OA medium at 25 °C, colonies were flat, medium dense, and aerial mycelium was rare in the central circular region and was white (7443); the reverse was similar to the front. After 14 days, colonies formed many dark green (378) spots, dense, with aerial mycelium distributed evenly; the reverse was similar to the front. After 31 days, colonies changed from white to gray (424); the reverse exhibited many small irregular brown (1615) areas. The average growth rate on OA medium was 10.3 mm/day.

Additional material studied—China, Yunnan Province, Menghai County, 21°55′25″ N, 100°35′41″ E, on decayed leaves, 18 March 2023, C.Z. Yin, Z.X. Zhang and X.G. Zhang, HSAUP 242405, living culture SAUCC242405.

Notes—the phylogenetic analysis of ITS, LSU, *rpb2*, and *tub2* sequences revealed a close relationship between *Daldinia menghaiensis*, *D. subvernicosa*, and *D. vernicosa*, and they are both on a branch with better support (MLBS/BPP = 99/1). *Daldinia menghaiensis* was different from *D. subvernicosa* by 25/464 bp in ITS, 16/1107 bp in LSU, 39/684 bp in *rpb2*, and 57/949 bp in *tub2* and different from *D. vernicosa* by 35/552 bp in ITS, 19/1226 bp in LSU, 54/867 bp in *rpb2*, and 62/1080 bp in *tub2*. Morphologically, compared with *Daldinia vernicosa*, the number of conidiogenous cells at the terminus of conidiophores in *D. menghaiensis* is less (1–2 vs. 1–4) and is generally long (16.9–23.5 vs. 8.0–23.0 µm), but the conidia are narrower (3.1–4.0 vs. 4.5–6.0 µm) [3]. Under the same conditions, the medium of *D. menghaiensis* always produces pigmentation, and mycelia become darker, but *D. subvernicosa* does not [7]. Therefore, *D. menghaiensis* can be identified as a new anamorphic species of *Daldinia* by phylogenetic and morphological comparison.

## 4. Discussion

This study investigated the fungal resources in five provinces in southern China, and the strains of *Daldinia* isolated in this survey were mainly collected from forests and mountainous areas, with altitudes ranging from 151.57 to 1876.7 m.a.s.l. These strains were found in all five provinces of southern China, which proved that they were also widely distributed in southern China. The number and types of strains were the highest in Yunnan Province, followed by Hainan Province, and the least were in Guizhou Province. The overall distribution of all strains in each province is shown in Figure 2a. We noticed that with similar sample sizes collected in each province, more strains of *Daldinia* were collected in the Yunnan and Hainan provinces in the tropical monsoon climate zone than in the other three provinces in the subtropical monsoon climate zone. Our preliminary hypothesis posits that these primary forests and mountains, boasting warm and humid climate conditions and abundant vegetation, may harbor more *Daldinia* as well as other fungal. The plant specimens collected in this survey were divided into diseased and decayed leaves, and 84 new host plants were determined by identifying the strains of *Daldinia*. They belong to 43 plant families, among which *Lauraceae* (10.84%), *Poaceae* (9.64%), *Moraceae* (6.02%), *Theaceae* (6.02%), and *Fagaceae* (4.82%) have a relatively high proportion, and there is little difference in the proportion of other families. The number of each plant family is shown in Figure 2b. In addition, dicots accounted for 84.34% of all plant hosts, while monocots were only *Poaceae*, *Zingiberaceae*, *Iridaceae*, and *Asparagaceae*. Therefore, it can be preliminarily inferred that the genus *Daldinia* does not exhibit strong specificity of host, and dicots account for the majority of hosts.

Since the establishment of the genus *Daldinia*, continuous advancements have been made in the methods utilized for its identification. Now, a widely accepted approach for identifying *Daldinia* involves phylogenetic analysis based on ITS, LSU, *rpb2*, and *tub2* combined with morphological comparison. A total of 104 records encompassing various species of *Daldinia* were retrieved from the Index Fungorum (https://www.indexfungorum.org/, accessed on 7 September 2024), among which most of the species with complete ITS, LSU, *rpb2*, and *tub2* sequences are included in Table 1. We also used this method to identify three known species (*Daldinia bambusicola*, *D. childiae*, and *D. eschscholtzii*) and seven new anamorphic species (*D. ehretiae*, *D. jianfengensis*, *D. ledongensis*, *D. menghaiensis*, *D. rhododendri*, *D. spatholobi*, and *D. thunbergiae*). In this study, we not only provided sequence information for new species but also perfected sequence data for known species, such as the *rpb2* and *tub2* sequence data of *D. childiae*, after species were confirmed through anamorph characteristics and multiple sequence alignments. The *Daldinia* strains were cultured using PDA and OA mediums, and the colony morphology at 7 days, 14 days, and 31 days of culture was observed and recorded, which could also assist in the identification of the strain while showing the colony changes. In the process of cultivating the strains of *Daldinia*, we also noticed an interesting phenomenon: taking the new species discovered this time as an example, whether on PDA medium or OA medium, *Daldinia* can be divided into two categories. One type produces a large number of spores and forms a regular large-area pigment precipitation area on the medium, and most of them form circular pigment precipitation around 2 weeks, such as *D. ehretiae*, *D. jianfengensis*, *D. menghaiensis*, *D. rhododendri*, *D. spatholobi*, and *D. thunbergiae*. And another type features the production of very small amounts of spores and the formation of small areas of pigment precipitation on the culture medium, such as *D. ledongensis*. Furthermore, it is worth noting that there have been numerous reports that *Daldinia* are endophytes, some even related to insects, and spores of *Daldinia* typically exhibit both teleomorph and anamorph modes, but the specimens obtained during this survey were found on diseased leaves and did not directly produce spores of teleomorph in their natural habitat. So, inducing *Daldinia* to produce spores of teleomorph in the laboratory presents an intriguing yet challenging task. At the same time, we will continue to conduct field collection work on teleomorphic species of *Daldinia* in the next step and improve the teleomorphic data of *Daldinia* found in this survey as soon as possible. Interestingly, due to the abundance of secondary metabolites within the genus *Daldinia*, there has been a growing trend towards utilizing HPLC profiling for metabolite analysis and incorporating chemical classification into the identification process. In their investigation of *Xylariales* in Thailand, Wongkanoun et al. employed morphological characters, phylogenetic analysis based on multi-locus sequences, and comprehensive analysis of secondary metabolites based on high-performance liquid chromatography-diode array detection and mass spectrometry, leading to the identification of three novel species, *D. flavogranulata*, *D. phadaengensis*, and *D. chiangdaoensis* [19]. Moving forward, we will continue conducting comprehensive research on resource exploration and metabolic characteristic analysis of the genus *Daldinia*.

## Figures and Tables

**Table 1:** Information on strains used in phylogenetic analysis of the genus *Daldinia*.

Species	Strains	Country	GenBank Accession Numbers				Reference
			ITS	LSU	*rpb2*	*tub2*	
*Annulohypoxylon annulatum*	CBS 140775	USA	KY610418	KY610418	KY624263	KX376353	[5]
*Annulohypoxylon moriforme*	CBS 123579	France	KX376321	KY610425	KY624289	KX271261	[5]
*Annulohypoxylon nitens*	MFLUCC 12.0823	Thailand	KJ934991	KJ934992	KJ934994	KJ934993	[18]
*Annulohypoxylon stygium*	MUCL 54601	French	KY610409	KY610475	KY624292	KX271263	[5]
*Annulohypoxylon truncatum*	CBS 140778	USA	KY610419	KY610419	KY624277	KX376352	[5]
*Daldinia andina*	CBS 114736 *	Ecuador	AM749918	KY610430	KY624239	KC977259	[5]
*Daldinia bambusicola*	CBS 122872 *	Thailand	KY610385	KY610431	KY624241	AY951688	[5]
*Daldinia bambusicola*	TBRC 8878	Thailand	MH922869	MH922870	MK165431	MK165422	[7]
*Daldinia bambusicola*	TBRC 8879	Thailand	MH922872	MH938543	MK165432	MK165423	[7]
*Daldinia bambusicola*	BCC27937	Thailand	MN153861	MN153876	MN172217	N/A	[19]
*Daldinia bambusicola*	BCC33678	Thailand	MN153860	MN153877	MN172218	N/A	[19]
***Daldinia bambusicola***	**SAUCC197001**	**China**	**PP145311**	**PP198902**	**PP263619**	**PP277065**	**This study**
*Daldinia brachysperma*	BCC33676	Thailand	MN153854	MN153871	N/A	MN172205	[19]
*Daldinia caldariorum*	MUCL 49211	France	AM749934	KY610433	KY624242	KC977282	[5]
*Daldinia caldariorum*	CBS122874	USA	KU683756	KU683756	KU684289	KU684128	[20]
*Daldinia chiangdaoensis*	BCC88220 *	Thailand	MN153850	MN153867	MN172208	MN172197	[19]
*Daldinia chiangdaoensis*	BCC88221	Thailand	MN153851	MN153868	MN172209	MN172198	[19]
***Daldinia childiae***	**SAUCC133401**	**China**	**PP145313**	**PP198904**	**PP263621**	**PP277067**	**This study**
*Daldinia concentrica*	CBS 113277	Germany	AY616683	KY610434	KY624243	KC977274	[5]
*Daldinia dennisii*	CBS 114741 *	Australia	JX658477	KY610435	KY624244	KC977262	[5]
*Daldinia eschscholtzii*	MUCL 45435	Benin	JX658484	KY610437	KY624246	KC977266	[5]
*Daldinia eschscholtzii*	TBRC 8876	Thailand	MH938532	MH938541	MK165429	MK165420	[7]
*Daldinia eschscholtzii*	BCC27887	Thailand	MN153861	MN153878	MN172214	N/A	[19]
*Daldinia eschscholtzii*	BCC28091	Thailand	MN153862	MN153879	MN172215	N/A	[19]
*Daldinia eschscholtzii*	BCC62428	Thailand	MN153863	MN153880	MN172216	N/A	[19]
***Daldinia eschscholtzii***	**SAUCC265301**	**China**	**PP145315**	**PP198906**	**PP263623**	**PP277069**	**This study**
***Daldinia ehretiae***	SAUCC228302 *	**China**	**PP145319**	**PP198888**	**PP263613**	**PP277051**	**This study**
***Daldinia ehretiae***	**SAUCC228303**	**China**	**PP145320**	**PP198889**	**PP263614**	**PP277052**	**This study**
*Daldinia flavogranulata*	BCC89363 *	Thailand	MN153856	MN153873	MN172211	MN172200	[19]
*Daldinia flavogranulata*	BCC89365	Thailand	MN153857	MN153874	MN172212	MN172201	[19]
*Daldinia flavogranulata*	BCC89376	Thailand	MN153858	MN153875	MN172213	MN172202	[19]
***Daldinia jianfengensis***	SAUCC373804 *	**China**	**PP145325**	**PP198890**	**PP263615**	**PP277053**	**This study**
***Daldinia jianfengensis***	**SAUCC373805**	**China**	**PP145326**	**PP198891**	**PP263616**	**PP277054**	**This study**
*Daldinia korfii*	EBS 067	Argentina	KY204018	N/A	N/A	KY204014	[21]
*Daldinia korfii*	EBS 473	Argentina	KY204020	N/A	N/A	KY204016	[21]
*Daldinia kretzschmarioides*	TBRC 8875	Thailand	MH938531	MH938540	MK165425	MK165416	[7]
***Daldinia ledongensis***	SAUCC393602 *	**China**	**PP145327**	**PP198892**	**N/A**	**PP277055**	**This study**
***Daldinia ledongensis***	**SAUCC393603**	**China**	**PP145328**	**PP198893**	**N/A**	**PP277056**	**This study**
*Daldinia loculatoides*	CBS 113279	UK	AF176982	KY610438	KY624247	KX271246	[5]
*Daldinia macaronesica*	CBS 113040	Spain	KY610398	KY610477	KY624294	KX271266	[5]
***Daldinia menghaiensis***	SAUCC242404 *	**China**	**PP145323**	**PP198894**	**PP263617**	**PP277057**	**This study**
***Daldinia menghaiensis***	**SAUCC242405**	**China**	**PP145324**	**PP198895**	**PP263618**	**PP277058**	**This study**
*Daldinia phadaengensis*	BCC89349 *	Thailand	MN153852	MN153869	MN172206	MN172195	[19]
*Daldinia phadaengensis*	BCC89350	Thailand	MN153853	MN153870	MN172207	MN172196	[19]
*Daldinia petriniae*	MUCL 49214	Austria	AM749937	KY610439	KY624248	KC977261	[5]
*Daldinia placentiformis*	MUCL 47603	Mexico	AM749921	KY610440	KY624249	KC977278	[5]
*Daldinia pyrenaica*	MUCL 53969	France	KY610413	KY610413	KY624274	KY624312	[5]
***Daldinia rhododendri***	SAUCC460001 *	**China**	**PP145330**	**PP198896**	**N/A**	**PP277059**	**This study**
***Daldinia rhododendri***	**SAUCC460002**	**China**	**PP145329**	**PP198897**	**N/A**	**PP277060**	**This study**
***Daldinia spatholobi***	SAUCC203501 *	**China**	**PP145318**	**PP198898**	**N/A**	**PP277061**	**This study**
***Daldinia spatholobi***	**SAUCC203502**	**China**	**PP145317**	**PP198899**	**N/A**	**PP277062**	**This study**
*Daldinia steglichii*	MUCL 43512	Papua New Guinea	KY610399	KY610479	KY624250	KX271269	[5]
*Daldinia subvernicosa*	TBRC 8877 *	Thailand	MH938533	MH938542	MK165430	MK165421	[7]
*Daldinia theissenii*	CBS 113044	Argentina	KY610388	KY610441	KY624251	KX271247	[5]
***Daldinia thunbergiae***	SAUCC228601 *	**China**	**PP145322**	**PP198900**	**N/A**	**PP277063**	**This study**
***Daldinia thunbergiae***	**SAUCC228602**	**China**	**PP145321**	**PP198901**	**N/A**	**PP277064**	**This study**
*Daldinia vernicosa*	CBS 119316	Germany	KY610395	KY610442	KY624252	KC977260	[5]
*Graphostroma platystomum*	CBS 270.87 *	France	JX658535	DQ836906	KY624296	HG934108	[5]
*Hypomontagnella monticulosa*	MUCL 54604	French	KY610404	KY610487	KY624305	KX271273	[5]
*Hypomontagnella monticulosa*	BCC58592	Thailand	MN153864	MN153881	MN172219	MN172204	[19]
*Hypomontagnella monticulosa*	BCC69203	Thailand	MN153865	MN153882	MN172220	MN172203	[19]
*Hypomontagnella submonticulosa*	CBS 115280	France	KC968923	KY610457	KY624226	KC977267	[5]
*Hypoxylon crocopeplum*	CBS 119004	France	KC968907	KY610445	KY624255	KC977268	[5]
*Hypoxylon fragiforme*	MUCL 51264	Germany	KC477229	KM186295	KM186296	KX271282	[5]
*Hypoxylon fuscum*	CBS 113049	France	KY610401	KY610482	KY624299	KX271271	[5]
*Hypoxylon haematostroma*	MUCL 53301	France	KC968911	KY610484	KY624301	KC977291	[5]
*Hypoxylon haematostroma*	BCC50533	Thailand	MN153866	MN153883	MN172221	N/A	[19]
*Hypoxylon investiens*	CBS 118183	Malaysia	KC968925	KY610450	KY624259	KC977270	[5]
*Hypoxylon lateripigmentum*	MUCL 53304 *	France	KC968933	KY610486	KY624304	KC977290	[5]
*Hypoxylon lenormandii*	CBS 119003	Ecuador	KC968943	KY610452	KY624261	KC977273	[5]
*Hypoxylon petriniae*	CBS 114746 *	France	KY610405	KY610491	KY624279	KX271274	[5]
*Hypoxylon rickii*	MUCL 53309	France	KC968932	KY610416	KY624281	KC977288	[5]
*Hypoxylon rubiginosum*	MUCL 52887	Germany	KC477232	KY610469	KY624266	KY624311	[5]
*Hypoxylon samuelsii*	MUCL 51843	France	KC968916	KY610466	KY624269	KC977286	[5]
*Jackrogersella cohaerens*	CBS 119126	Germany	KY610396	KY610497	KY624270	KY624314	[5]
*Jackrogersella minutella*	CBS 119015	Portugal	KY610381	KY610424	KY624235	KX271240	[5]
*Jackrogersella multiformis*	CBS 119016	Germany	KC477234	KY610473	KY624290	KX271262	[5]
*Pyrenopolyporus hunteri*	MUCL 52673	Ivory Coast	KY610421	KY610472	KY624309	KU159530	[5]
*Pyrenopolyporus laminosus*	MUCL 53305 *	France	KC968934	KY610485	KY624303	KC977292	[5]
*Pyrenopolyporus laminosus*	TBRC 8871	Thailand	MH938527	MH938536	MK165424	MK165415	[7]
*Pyrenopolyporus laminosus*	BCC89383	Thailand	MN153855	MN153872	MN172210	MN172199	[19]
*Pyrenopolyporus nicaraguensis*	CBS 117739 *	Burkina Faso	AM749922	KY610489	KY624307	KC977272	[5]
*Pyrenopolyporus symphyon*	TBRC 8873	Thailand	MH938529	MH938538	MK165428	MK165419	[7]
*Xylaria hypoxylon*	CBS12260	Sweden	KY610407	KY610495	KY624231	KX271279	[5]

Notes: The new strains for phylogenetic analysis introduced in this experiment are shown in bold. Ex-type or ex-epitype strains are marked with an “*”. N/A: Not available.

**Table 2:** Collection information of *Daldinia* specimens.

Species	Collection Location	Collection Time	Strains	Host	Illustration
*Daldinia bambusicola*	Yunnan Province, Jinghong City, Xishuangbanna primitive forest Park, 22°1′52″ N, 100°52′36″ E	17 March 2023	SAUCC197001	*Viburnum rhytidophyllum*	Figure 3a
			SAUCC203501	*Spatholobus suberectus*	Figure 4a
			SAUCC204601	*Piper Nigrum*	Figure 4b
			SAUCC206901	*Cinnamomum verum*	Figure 4g
	Yunnan Province, Menghai County, Nanuo Mountain, 21°55′25″ N, 100°35′41″ E	18 March 2023	SAUCC240501	*Koelreuteria paniculata*	Figure 4c
	Hainan Province, Lingshui County, Diaoluo Mountain, 18°41′45″ N, 109°56′26″ E	8 April 2023	SAUCC283401	*Ficus hirta*	Figure 4d
	Hainan Province, Ledong County, Jianfengling National Forest Park, 18°41′18″ N, 108°51′31″ E	12 April 2023	SAUCC392801	*Schima superba*	Figure 4e
	Sichuan Province, Chengdu City, Dujiangyan, 30°59′58″ N, 108°51′31″ E	24 June 2023	SAUCC423801	*Citrus maxima*	Figure 4f
	Sichuan Province, Leshan City, 29°34′56″ N, 103°17′20″ E	25 June 2023	SAUCC433001	*Phyllostachys heteroclada*	Figure 4h
	Sichuan Province, Liangshan Prefecture, Xichang City, 27°45′29″ N, 102°18′31″ E	2 July 2023	SAUCC518401	*Ageratina adenophora*	Figure 4i
	Guizhou Province, Qiandongnan Prefecture, Majiang County, Gudong Town, 26°25′5″ N, 107°21′18″ E	22 August 2023	SAUCC551401	*Lophatherum gracile*	Figure 4j
	Guizhou Province, Qiannan Prefecture, Sandu County, 25°55′52″ N, 107°57′34″ E	24 August 2023	SAUCC570801	*Ulmus pumila*	Figure 4k
*Daldinia childiae*	Fujian Province, Wuyishan City, Xingcun Town, 27°45′5″ N, 117°41′3″ E	15 October 2022	SAUCC133401	*Machilus nanmu*	Figure 5a
			SAUCC148701	*Eurya japonica*	Figure 6a
	Yunnan province, Jinghong City, Xishuangbanna primitive Forest Park, 22°1′52″ N, 100°52′36″ E	17 March 2023	SAUCC209301	*Piper nigrum*	Figure 6e
	Yunnan Province, Jinghong City, Binjiang Avenue, 22°0′41″ N, 100°48′15″ E	20 March 2023	SAUCC220001	*Microstegium vimineum*	Figure 6b
	Hainan Province, Lingshui County, Benhao Town, 18°41′54″ N, 109°52′51″ E	9 April 2023	SAUCC314401	*Pseudosasa japonica*	Figure 6f
	Hainan Province, Ledong County, Jianfengling National Forest Park, 18°42′35″ N, 108°52′35″ E	12 April 2023	SAUCC387901	*Litsea cubeba*	Figure 6c
			SAUCC388601	*Quercus glauca*	Figure 6d
			SAUCC392901	*Schima superba*	Figure 6i
			SAUCC397101	*Castanopsis calathiformis*	Figure 6g
	Sichuan Province, Leshan City, Erhong Road, 29°35′31″ N, 103°22′39″ E	25 June 2023	SAUCC439301	*Symplocos sumuntia*	Figure 6h
*Daldinia eschscholtzii*	Hainan Province, Sanya City, Jiyang District, Dongtian Ridge, 18°23′35″ N, 109°38′14″ E	8 April 2023	SAUCC265301	*Lysimachia clethroides*	Figure 7a
	Fujian Province, Wuyishan City, Xingcun Town, 27°44′47″ N, 117°40′36″ E	15 October 2022	SAUCC117601	*Machilus thunbergii*	Figure 8(a1)
			SAUCC123301	*Lindera aggregata*	Figure 8(a2)
			SAUCC132901	*Maesa japonica*	Figure 8(a3)
	Yunnan Province, Xishuangbanna Tropical Botanical Garden, 21°55′52″ N, 101°14′50″ E	15 March 2023	SAUCC179601	*Gonocaryum lobbianum*	Figure 8(b1)
			SAUCC179701	*Engelhardia spicata*	Figure 8(b2)
			SAUCC182201	*Artocarpus heterophyllus*	Figure 8(b3)
			SAUCC184501	*Tabernaemontana divaricata*	Figure 8(b4)
			SAUCC187501	*Piper nigrum*	Figure 8(b5)
			SAUCC191301	*Ficus tinctoria*	Figure 8(b6)
			SAUCC191401	*Lycianthes biflora*	Figure 8(b7)
			SAUCC192301	*Bischofia javanica*	Figure 8(b8)
	Yunnan province, Jinghong City, Xishuangbanna primitive Forest Park, 22°1′52″ N, 100°52′36″ E	17 March 2023	SAUCC197301	*Artocarpus hypargyreus*	Figure 8(c1)
			SAUCC201001	*Murraya exotica*	Figure 8(c2)
			SAUCC205701	*Fargesia spathacea*	Figure 8(c3)
			SAUCC206001	*Zingiber zerumbet*	Figure 8(c4)
			SAUCC206301	*Microstegium vimineum*	Figure 8(c5)
			SAUCC215701	*Kadsura longipedunculata*	Figure 8(c6)
	Yunnan Province, Xishuangbanna Prefecture, Jinghong City, 22°0′41″ N, 100°48′15″ E	20 March 2023	SAUCC217201	*Iris tectorum*	Figure 8(d3)
			SAUCC217901	*Cordyline fruticosa*	Figure 8(d2)
			SAUCC218101	*Graptophyllum pictum*	Figure 8(d1)
			SAUCC220401	*Calliandra haematocephala*	Figure 8(d4)
			SAUCC220501	*Ficus subulata*	Figure 8(d5)
	Yunnan Province, Menghai County, Nanuo Mountain, 21°55′25″ N, 100°35′41″ E	18 March 2023	SAUCC231401	*Castanopsis calathiformis*	Figure 8(e1)
			SAUCC237801	*Betula utilis*	Figure 8(e2)
			SAUCC239601	*Ageratina adenophora*	Figure 8(e3)
			SAUCC243701	*Dendrocalamus latiflorus*	Figure 8(e4)
			SAUCC244401	*Tithonia diversifolia*	Figure 8(e5)
	Hainan Province, Lingshui County, Diaoluo Mountain, 18°43′35″ N, 109°52′1″ E	9 April 2023	SAUCC296301	*Mangifera indica*	Figure 8(f1)
			SAUCC297601	*Lindera nacusua*	Figure 8(f2)
	Hainan Province Lingshui County, Benhao town, 18°41′54″ N, 109°52′51″ E	9 April 2023	SUACC300301	*Syzygium levinei*	Figure 8(g1)
			SAUCC309901	*Pseudosasa japonica*	Figure 8(g3)
			SAUCC311501	*Eurya groffii*	Figure 8(g2)
			SAUCC314101	*Exbucklandia populnea*	Figure 8(g4)
			SAUCC314201	*Bridelia balansae*	Figure 8(g5)
	Hainan Province, Baoting County, Baocheng town, 109°41′35″ N, 18°42′6″ E	10 April 2023	SAUCC323201	*Pronephrium gymnopteridifrons*	Figure 8h
	Hainan Province, Changjiang County, Qicha Town, 19°7′2″ N, 109°9′1″ E,	11 April 2023	SAUCC331301	*Parashorea chinensis*	Figure 8i
	Hainan Province, Changjiang County, Bawangling National Forest Park, 19°7′17″ N, 109°7′6″ E	11 April 2023	SAUCC342001	*Clematis uncinata*	Figure 8(j1)
			SAUCC343301	*Diplospora dubia*	Figure 8(j2)
			SAUCC364301	*Tilia cordata*	Figure 8(j3)
			SAUCC367501	*Triadica cochinchinensis*	Figure 8(j4)
	Hainan Province, Ledong County, Jianfeng town, 18°42′35″ N, 108°52′35″ E	12 April 2023	SAUCC367701	*Rhaphiolepis indica*	Figure 8(k1)
			SAUCC369301	*Dryopteris podophylla*	Figure 8(k2)
			SAUCC371901	*Chengiodendron matsumuranum*	Figure 8(k3)
			SAUCC373001	*Schima superba*	Figure 8(k4)
			SAUCC373901	*Camellia oleifera*	Figure 8(k5)
			SAUCC374001	*Polyspora chrysandra*	Figure 8(k6)
			SAUCC374501	*Lithocarpus henryi*	Figure 8(k7)
			SAUCC374601	*Eurya nitida*	Figure 8(k8)
			SAUCC375901	*Elaeocarpus decipiens*	Figure 8(k9)
			SAUCC376101	*Meliosma rigida*	Figure 8(k10)
			SAUCC376901	*Rhododendron latoucheae*	Figure 8(k11)
			SAUCC377201	*Litsea cubeba*	Figure 8(k12)
	Hainan Province, Ledong County, Jianfengling National Forest Park, 18°41′18″ N, 108°51′31″ E	12 April 2023	SAUCC394901	*Psidium cattleyanum*	Figure 8l
	Sichuan Province, Chengdu City, Dujiangyan, 30°59′58″ N, 108°51′31″ E	24 June 2023	SAUCC424501	*Phoebe zhennan*	Figure 8(m1)
			SAUCC426101	*Rhus chinensis*	Figure 8(m2)
	Sichuan Province, Yaan City, Tianquan County, 30°0′21″ N, 102°30′25″ E	26 June 2023	SAUCC440101	*Machilus nanmu*	Figure 8n
	Guizhou Province, Qiannan Prefecture, Pingtang County, 25°47′42″ N, 107°23′10″ E	23 August 2023	SAUCC561601	*Liquidambar formosana*	Figure 8o
*Daldinia ehretiae*	Yunnan Province, Jinghong City, Sancha River, 22°10′10″ N, 100°51′49″ E	19 March 2023	SAUCC228302	*Ehretia acuminata*	Figure 9a
*Daldinia rhododendri*	Yunnan Province, Diqing Prefecture, Shangri-la City, 27°58′43″ N, 99°34′24″ E	28 June 2023	SAUCC460001	*Rhododendron decorum*	Figure 10a
*Daldinia spatholobi*	Yunnan Province, Jinghong City, Xishuangbanna primitive Forest Park, 22°1′52″ N, 100°52′36″ E	17 March 2023	SAUCC203501	*Spatholobus suberectus*	Figure 11a
*Daldinia thunbergiae*	Yunnan Province, Jinghong City, San-cha River, 22°10′10″ N, 100°51′49″ E	19 March 2023	SAUCC228601	*Thunbergia grandiflora*	Figure 12a
*Daldinia jianfengensis*	Hainan Province, Ledong County, Jianfeng Town, 18°41′19″ N, 108°51′31″ E	12 April 2023	SAUCC373804	decayed leaves	
*Daldinia ledongensis*	Hainan Province, Ledong County, 18°41′18″ N, 108°51′31″ E	12 April 2023	SAUCC393602	decayed leaves	
*Daldinia menghaiensis*	Yunnan Province, Menghai County, 21°55′25″ N, 100°35′41″ E	18 March 2023	SAUCC242404	decayed leaves	

## Data Availability

All datasets in this study are included in this article/Supplementary Materials.

## Supplementary Materials

The following supporting information can be downloaded at: https://www.mdpi.com/article/10.3390/jof10100700/s1, Table S1: PCR primer sequence and reaction conditions used in this experiment.
