# Supplementary material for: A Taxonomic and Phylogenetic Study of Anamorphic Strains of Daldinia (Hypoxylaceae, Xylariales) in Southern China
Source: J Fungi (Basel). 2024 Oct 7;10(10):700. doi: 10.3390/jof10100700 (PMC11508840; doi:10.3390/jof10100700)
Supplement: Supplementary file 1 [file jof-10-00700-s001.zip › jof-3149162-supplementary.pdf]

Table 1. PCR primer sequence and reaction conditions used in this experiment.

Loci	PCR primers	Sequence (5′ – 3′)	PCR cycles	References
ITS	ITS5	GGA AGT AAA AGT CGT AAC AAG G	35 cycles: 30 s at 95 °C, 30 s at 55 °C, 1 min at 72 °C	[14]
	ITS4	TCC TCC GCT TAT TGA TAT GC		
LSU	LR0R	GTA CCC GCT GAA CTT AAG C	35 cycles: 50 s at 95 °C, 30 s at 47 °C, 1 min 30 s at 72 °C	[14,15]
	LR7	TAC TAC CAC CAA GAT CT		
RPB2	fRPB2-5F	GAY GAY MGW GAT CAY TTY GG	35 cycles: 1 min at 95 °C, 2 min at 55 °C, increase of 1 °C / 5 s to 72 °C, 2 min at 72 °C	[16]
	fRPB2-7cR	CCC ATW GCY TGC TTM CCC AT		
TUB2	T1	AAC ATG CGT GAG ATT GTA AGT	35 cycles: 35 s at 94 °C, 55 s at 52 °C, 2 min at 72 °C	[17]
	T22	TCT GGA TGT TGT TGG GAA TCC		
